# A Time-Domain Model for the Generation of Spontaneous Otoacoustic Emissions in Resonant Cavities

**DOI:** 10.1007/s10162-026-01063-x

**Published:** 2026-07-10

**Authors:** Hero P. Wit

**Affiliations:** Department of Otorhinolaryngology/Head and Neck Surgery, University of Groningen, University Medical Center Groningen, Groningen, The Netherlands

**Keywords:** Self-sustaining oscillator, Micro-mechanical element, Cochlear amplifier, Standing wave, Fluid coupling, Frequency plateaus, Resonant cavity

## Abstract

A long-standing question in hearing science is whether spontaneous otoacoustic emissions (SOAEs) arise from a specific active site on the basilar membrane—they have a local origin—or whether they derive from distributed active elements working together in a resonant cavity—a global picture. This paper uses numerical modelling to investigate the difference between local and global activity in the ear. The cochlea is modelled as a graded array of discrete oscillators, each of fixed natural frequency and coupled only by the surrounding fluid. We examine what happens when the damping parameters of the system are varied in particular ways. Do the continuous oscillations that emerge at the middle ear carry the tell-tale frequency of a specific oscillator—pointing to a local origin—or is the oscillation one that arose from the global activity of multiple interacting oscillators? The basic oscillating element considered here is made up of two feedback-coupled masses, a general arrangement first proposed by Neely and Kim. An important parameter is the amount of undamping of the element, such that high undamping (low damping) energizes the system and tends to generate spontaneous oscillations. We use the time-domain state-space formalism to describe the interacting system of oscillating elements. We find that the entire system can be made local or global depending on the size and distribution of the undamping parameters. Thus, if a single element is made to continuously oscillate by increasing its undamping, a spontaneous otoacoustic emission (SOAE) will emerge at the oscillator’s natural frequency—it can be considered to have a local origin. On the other hand, if all the elements are provided with sufficient undamping, an SOAE can again emerge even though each individual element remains below its oscillation threshold—the conclusion being that the oscillation now arises from global coupling. In this manner, our numerical modelling shows that even when undamping is supplied irregularly all along the array (but not enough to make any individual element continuously oscillate), one or more SOAEs can still be produced. When such a system is examined, we see an underlying pattern of standing waves, but even so, we see no clear evidence of a more or less regular process for the reflection of waves from irregularities at the apical side of a resonant cavity—which is what the generally accepted global model of SOAE generation by coherent reflection calls for.

## Introduction

**Fig. 1:**
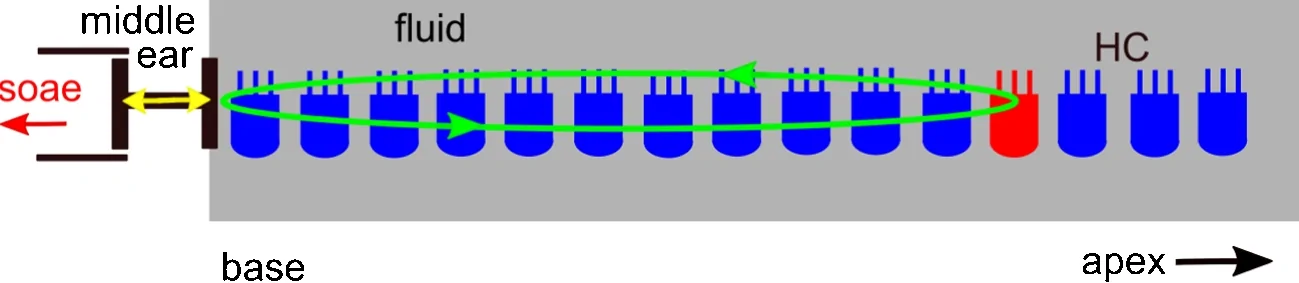
Schematic of the human inner ear, being a slightly adapted version of Fig. 3 in Shera’s paper on the difference between a local and a global system for the generation of a single SOAE. HC, hair cells. The red hair cell is at the peak of the travelling wave where reflection is said to occur. Another reflection occurs at the middle ear, and so that a standing wave appears as the sum of forward and backward travelling waves, shown in green

Otoacoustic emissions were discovered almost 50 years ago by Kemp [1, 2] as “evoked cochlear mechanical responses” (ECMRs). Even when no evoking stimulus is present, weak tones can be detected emerging continuously from the inner ears of frogs, lizards, birds, and mammals—so-called spontaneous otoacoustic emissions (SOAEs).

Two broad classes of models have been used to understand the generation of SOAEs. At one extreme, there are “local” models, where isolated micro-mechanical elements permanently oscillate, independently of the other elements. At the other, there are “global” models, in which none of the elements oscillates in isolation, but when coupled by the inner ear fluid, the system produces one or more self-sustaining oscillations. The difference between the two classes, and the implications for how SOAEs are generated, was a few years ago extensively described by Shera [3].

Figure 1 is a slightly adapted version of Fig. 3 in Shera’s 2022 [3] paper. It illustrates how an SOAE is generated by the human ear, functioning as a global oscillator. The red hair cell borders a “resonant cavity.”

Shera [3] describes the operation of the global oscillator in Fig. 1 below as follows: “The region between the stapes and the peak of the traveling wave (the red hair cell in Fig. 1) acts as a “resonant cavity” enclosing a non-linear gain medium powered by hair cells. Partial reflection of forward- and backward-traveling waves (the green line in Fig. 1) occurs at each end of the cavity. Standing waves occur at frequencies where the round-trip phase changes by an integral number of cycles. Standing-wave amplitudes are stabilized when the round-trip gain matches the losses due to internal damping and emission into the ear canal.”

Examples of local models are given in [4–10]. A notable feature of all these local models is that self-sustaining oscillators cluster in frequency plateaus [11], and each plateau is taken to being the source of an SOAE (strictly speaking, these models, except [7], do not generate SOAEs, because they lack a connection to the tympanic membrane).

An earlier effort to illustrate the similarities and differences between the two classes of model was set out in the *intermezzi* of an earlier paper [9]. The present contribution is a continuation of that earlier work, and here the characteristics of the two classes of systems are investigated and illustrated in more detail. We find that the difference between them can be simply categorized in terms of where and how undamping—the source of the SOAEs energy—is applied within an array of micro-mechanical elements. If undamping, large enough to make the system oscillate continuously at some particular frequency, is applied to just one oscillator in the array, the signal detected in the ear canal will carry its signature frequency, conclusively indicating that the SOAE has a local origin. On the other hand, even if the undamping applied to each of the elements is not large enough to make any one of them oscillate, the broader system can still continuously oscillate if an irregularity is introduced into the larger set of individual undamping values—as we will show later. In such a case, the SOAE can be said to have a global origin.

In more detail, our modelling will also compare the situation in which the array of micro-mechanical elements has one isolated irregularity in its undamping parameters with the situation where the undamping parameters are irregular all along the array. In these two situations, the underlying mechanisms that create continuous oscillations are compared and explored. Of particular interest, we search here for “coherent reflection,” which is a key component in the global standing wave paradigm [3, 12–14].

The micro-mechanical elements we use to investigate the two classes of behavior are based on a pair of actively coupled masses, a configuration first proposed by Neely and Kim [15]. Our cochlear model comprises an extended array of such pairs.

To keep the presentation clear and comprehensible, details of this system are placed in Appendices 1 and 3.

If a global oscillator is defined as a system of two or more oscillators that do not oscillate in isolation, but undergo a self-sustaining oscillation when coupled, then the system in Appendix 1 can be regarded as an example of an elementary global oscillator.

## The Model

**Fig. 2:**
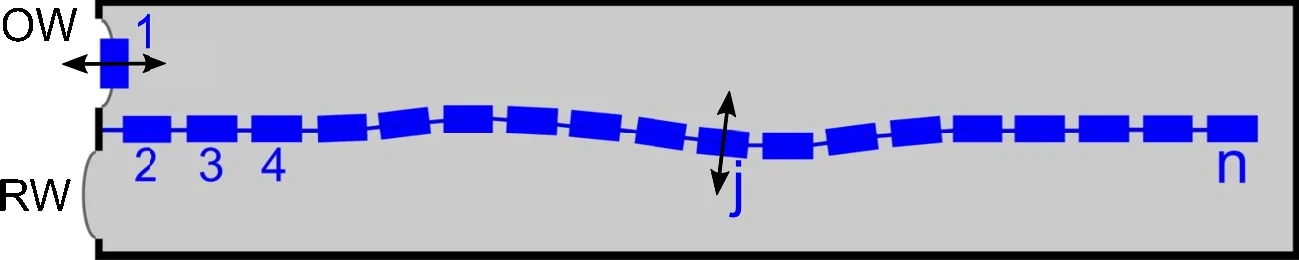
Schematic representation of a fluid-filled cochlea. **1** Stapes (and connected middle ear, considered to have a single resonant frequency); **2...n** Micro-mechanical elements, representing the sensing cells of the organ of Corti; **OW** oval window; **RW** round window; the lines between the elements represent the basilar membrane separating the two fluid compartments. In this model, each element is driven by fluid pressure only, and—as a consequence—the only coupling between the elements is via the fluid

Figure 2 is a schematic representation of the cochlea as a one-dimensional array of micro-mechanical elements in a fluid-filled box.

To calculate the dynamics of the entire fluid-coupled system, we solve the state-space equations set out 20 years ago in Elliott et al. [16]. When the solutions reveal continuous oscillation of oscillator 1 (the stapes and entire middle ear), then this shows the presence of an SOAE exiting the inner ear into the external ear canal.

Following Elliott et al. [16], the micro-mechanical elements *j* in Fig. 2 are chosen to be Neely & Kim elements [15], as investigated in Appendix 1. Importantly, note that there is no elastic and/or dissipative coupling between the elements (as there is, for instance, in the similar model of Fruth et al. [17]). The only coupling here is through the fluid.

In the Elliott model, a one-dimensional wave propagates along the *z*-axis of the cochlea according to the equation $\frac{\delta ^2p(t)}{\delta z^2}-\frac{2\rho }{H}$ $\ddot{z}(t)=0$, where *p*(*t*) is the waveform of the pressure difference across the cochlear partition; $\ddot{z}(t)$ is the radially averaged transverse acceleration of the cochlear partition; ρ is the density of the cochlear fluids, and *H* is the height of the canal above and below the cochlear partition, which is assumed to be constant (equation 3 in [16], equation 1 in [17]) (all units, unless otherwise stated, are SI units).

The equations to be solved for each element *j* ( for j=2 to *n*), for displacements $x_j(t)$ and $y_j(t)$ of masses $M_1$ and $M_2$ respectively, are adapted from equations (30, 31, 33) in [16] and (A1, A2) in [18], so that:

1 $$\begin{aligned} M_1 \ddot{x}_j(t)= &   a11_j\dot{x}_j(t)+a12_jx_j(t)+a13_j\dot{y}_j(t)\nonumber \\  &   +a14_jy_j(t)+p_{d,j}(t) \end{aligned}$$

2 $$\begin{aligned} M_2 \ddot{y}_j(t)= a21_j\dot{x}_j(t)+a22_jx_j(t)+a23_j\dot{y}_j(t)+a24_jy_j(t). \end{aligned}$$

Equations for a11, a12,..., a23, a24 in Appendix 3 include a saturating nonlinearity, for which the saturation parameter $s_m$ was given the (arbitrary) value 0.001 in all calculations (see Appendix 3). In addition, $p_{d,j}(t)$ is the force acting on element *j*, generated by the difference in fluid pressure across it; how its value is calculated is also set out in Appendix 3.

As an elementary representation of the middle ear and ear canal, an equation for oscillator 1 in Fig. 2 was added to Eqs. 1 and 2, so that

$$\ddot{x}_1(t)+d_1\omega _1\dot{x}_1(t)+\omega _1^2x_1(t)=a_1(t)+a_{ext}(t)$$ (3)

This is the equation for a harmonic oscillator with angular frequency $\omega _1$ and damping ratio $d_1$, while $a_1(t)$ is the acceleration of oscillator 1 generated by the inner ear fluid pressure, and $a_{ext}(t)$ the acceleration generated by an external force.

As a first step to check the model, the set of equations (1,2,3) was solved for an array of 350 micro-mechanical elements, for $a_{ext}(t) = \sin \omega _st$, where $\omega _s= 2\pi \times 3.12$ kHz is the angular frequency of the sustained oscillation (approximately the frequency of the SOAE in the next sections).

The values for the stiffness parameters $K_j$ and the damping parameters $C_j$ for each of the elements *j*, leading to natural frequencies of the elements decaying exponentially from 20 to 0.12 kHz along the array, are given in Appendix 3.

Other values were: $$M_1=0.044, M_2=0.0073$$, basilar membrane to inner hair cell lever gain g=1, damping $$d_1=0.1$$, angular frequency $$\omega _1=2000\pi $$ (natural frequency $$f_1= 1$$ *kHz*), and saturation value $$x_m=10^{-3} $$.

An important parameter is $\gamma _{0,j}$ in the equations for *a*11 and *a*12. This parameter controls the damping of the vibration of all the 350 masses $M_1$. The *larger* the value of $\gamma _0$, the *smaller* the damping, and here it was given the value 0.4 for all 350 micro-mechanical elements. This choice means that in the absence of fluid coupling (α=0) the impulse responses of all elements are damped oscillations (so there is no sustained activity).

The set of equations (1, 2, 3) was solved with the NDSolve routine in Wolfram 14.3 for desktop, for a total time of 100 ms and a time step of 10 μs. Initial values were $x_j(0)=\dot{x}_j(0)=y_j(0)=\dot{y}_j(0)=0$.

**Fig. 3:**
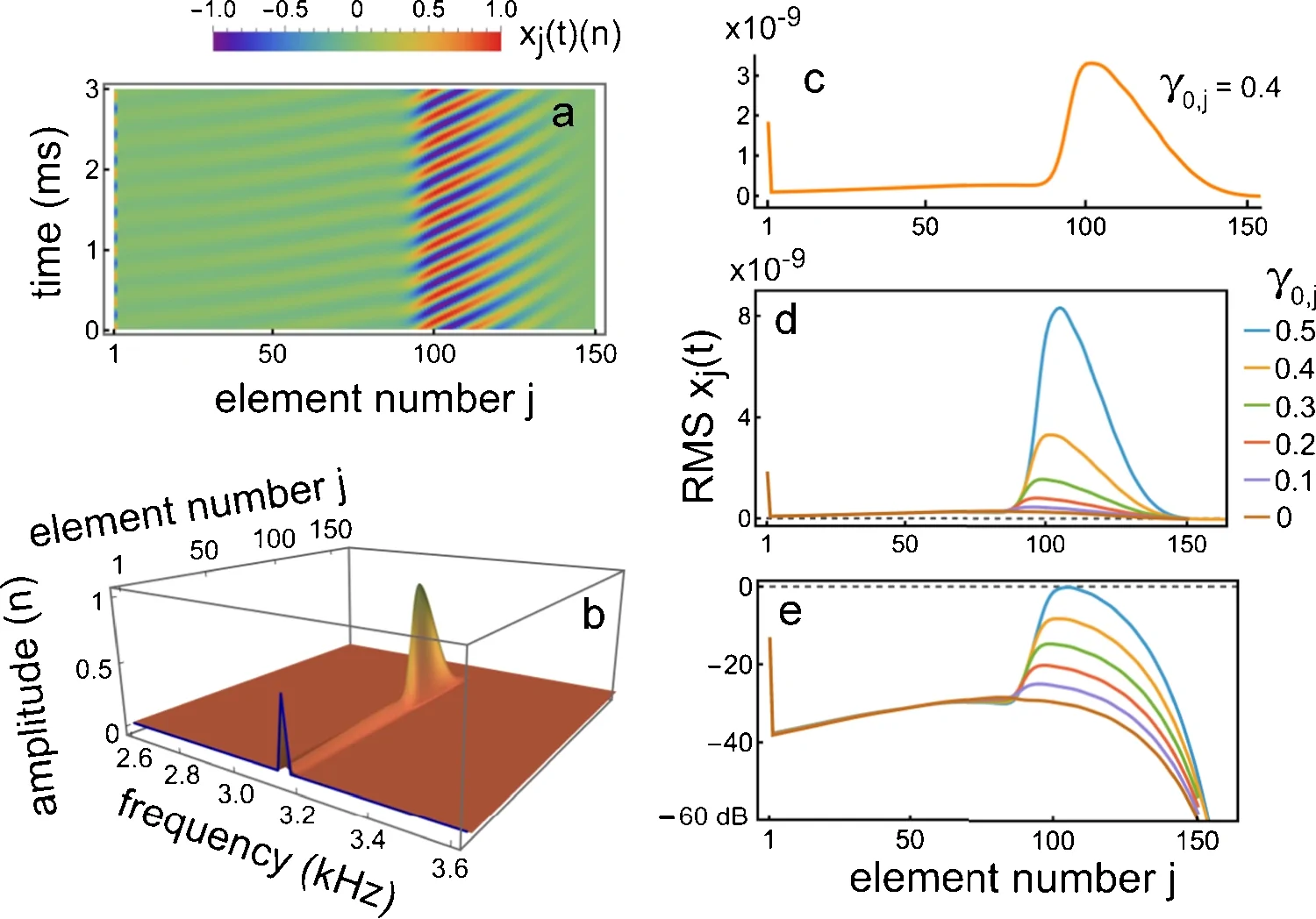
Calculated oscillations of the micro-mechanical elements in Fig. 2, if oscillator 1 (the middle ear) is driven by a sine wave of 3.12 kHz. **a** Density plot for the last 3 ms of the calculated 100 ms of the displacements $x_j(t)$. **b** 3D-plot for the frequency spectra for oscillator 1 and elements 2 to 160. **c** RMS values for $\gamma _{0,j}=0.4$ for displacements $x_j(t)$ for elements 1 to 155. **d** As c, but for six different values of $\gamma _0$. **e** As d, but with a logarithmic vertical scale

Results are shown in Fig. 3.

## A Single Global Oscillator

The calculations to obtain Fig. 3 were repeated so as to investigate what happens when there is no external forcing but there is a moderate level of undamping in the array of micro-mechanical elements. So $a_{ext}(t)=0$ and the initial values were: $x_j(0)=0, \dot{x}_j(0)=0$, for all *j*; $y_j(0)=0, \dot{y}_j(0)=1$, for j=2 to *n*. Parameter $\gamma _{0,j}$, that controls the damping, was given a value 0.4 for all micro-mechanical elements, except, crucially, for element 100, where $\gamma _{0,100}=0.6$. This means that element 100—when in isolation—will behave as a damped oscillator, and indeed for α=0 (no fluid coupling) the impulse responses of all elements were damped oscillations. Fluid pressure was calculated with equation 6 in Appendix 3.

**Fig. 4:**
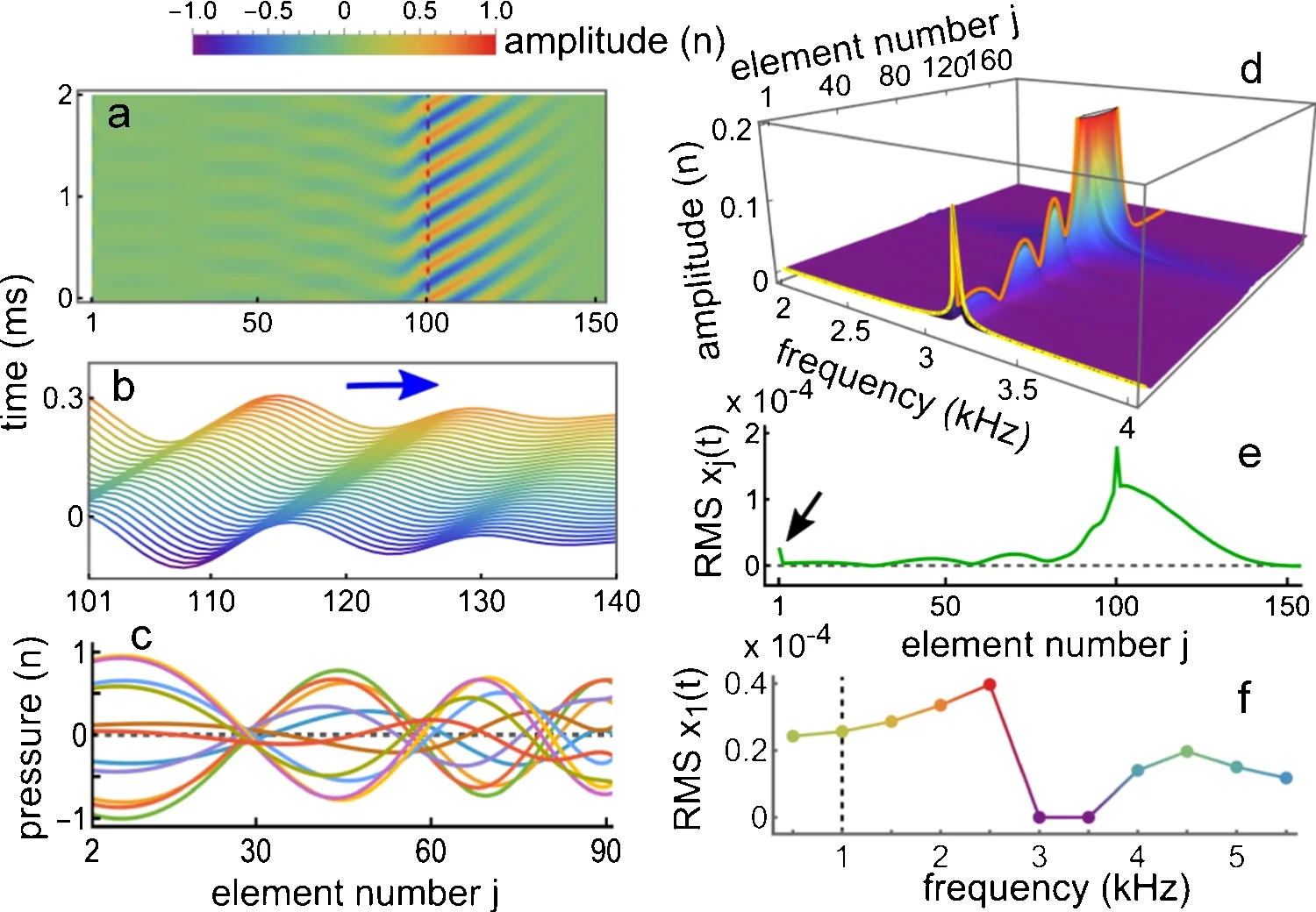
Paradigm of a global oscillator in which all the micro-mechanical elements are passive when working alone (low overall damping with $\gamma _{0,j}=0.4$, except for one irregularity with $\gamma _{0,100}=0.6$) but can continuously oscillate when coupled together. **a** Density plot for the last 2 ms of the calculated 100 ms of the displacements $x_j(t)$, for j=1 to 150 **b** Successive displacement profiles for elements 101 to 140 during one period in a waterfall plot, showing a damped wave travelling in the direction of the arrow. **c** Successive fluid pressure profiles along the array of elements during the last calculated period of oscillation, showing an evident standing wave pattern. **d** Frequency spectra for the last 50 ms of the calculated 100 ms of the displacements $x_j(t)$ in a 3D-plot, including for oscillator 1 (yellow line at 3.14 kHz), and for elements 2 to 185. The spectra around element 100 are truncated to better show the nodes and antinodes of the standing wave (the amplitude is normalized to 1 for element 100). **e** RMS values for the last 2 ms of the calculated 100 ms of the displacements $x_j(t)$. **f** RMS value for the last 50 ms of the calculated 100 ms of the displacement $x_1(t)$, which is the generated SOAE, calculated for 11 values of the natural frequency of oscillator 1. The dashed line marks the element indicated with an arrow in panel **e**

Results are shown in Fig. 4, which shows that element 100 introduces active behavior reminiscent of an SOAE (even though, for a single isolated oscillator, $\gamma _0$ needs to be larger than 1.06 to produce self-sustaining oscillation).

Thus, oscillator 1 and all elements with j<150 in Fig. 4 are seen to produce a self-sustaining oscillation, all with the same frequency, reflecting the generation of a single SOAE with this frequency (the “resonant cavity” in this case is between oscillator 1 and element 100). Because all elements behave as damped oscillators when not coupled by the fluid, the results shown in Fig. 4 can be said to be those for a global system, as illustrated by Shera [3] in his Fig. 3 (see also Fig. 1 here).

Although it may be surprising that a self-sustaining single frequency oscillation can arise in a nonlinear time-domain state-space model due to a single irregularity in an array of feedback gain parameters, this was in fact demonstrated two decades ago by Elliott et al. [16] in their Fig. 14. In their case, the irregularity was a single step from γ=0.850 down to γ=0.849, while the frequency of the self-sustaining oscillation was “very similar to the characteristic frequency at the position of the step.” In our situation, the frequency of the impulse response for element 100, for no fluid coupling and $\gamma _{0,100} =0.6$ is 3.10 kHz. This is to be compared with 3.14 kHz for the elements in Fig. 4.

It is of interest to note that Vignali [18] repeated the calculation that was used to obtain Fig. 14 in Elliott et al. [16], and that his figure 6.3, with a standing wave pattern, is directly comparable with our Fig. 4a (the standing wave pattern in Vignali’s figure is more clearly visible, because he calculated BM velocity, not displacement).

It should further be noted that the single irregularity in Elliott et al. [16] (see above), and also in Vignali [18], is a single step in an array of γ-values. Whereas in our case, it is a single anomaly of γ. Our case can be interpreted as two steps: one step up, followed by one step down.

## A Single Local Oscillator

**Fig. 5:**
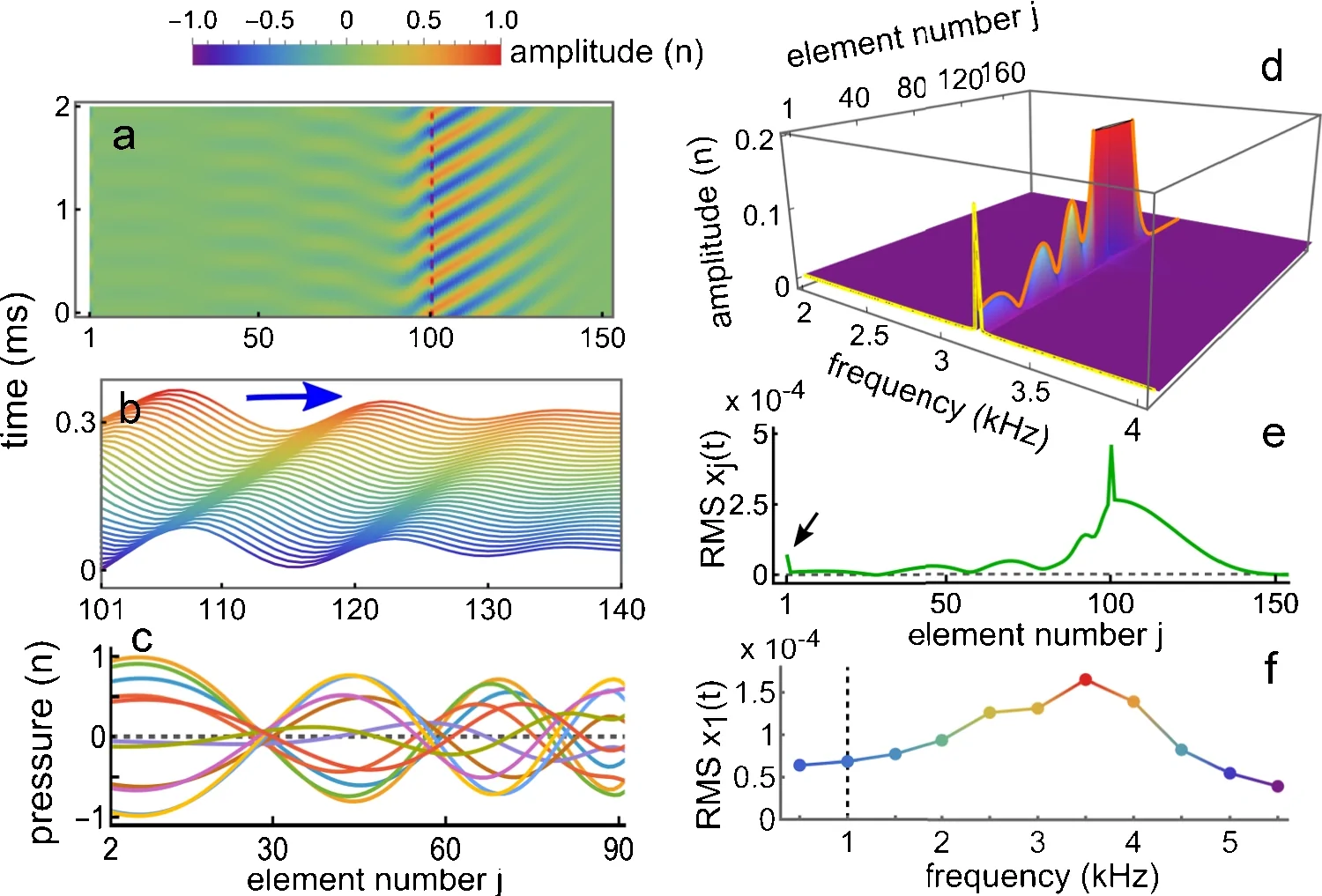
Paradigm of a system in which nearly all the micro-mechanical elements are passive when working alone (low overall damping with $\gamma _{0,j}=0.4$), except for element 100 being active with $\gamma _{0,100}=1.1$. **a** Density plot for the last 2 ms of the calculated 100 ms of the displacements $x_j(t)$, for j=1 to 150 **b** Successive displacement profiles for elements 101 to 140 during one period in a waterfall plot, showing a damped wave travelling in the direction of the arrow. **c** Successive fluid pressure profiles along the array of elements during the last calculated period of oscillation, showing a standing wave. **d** A 3D-plot of frequency spectra for the last 50 ms of the calculated 100 ms of the displacements $x_j(t)$, for oscillator 1 (yellow line at 3.14 kHz), as well as for elements 2 to 185. The spectra around element 100 are truncated to better show the nodes and antinodes of the standing wave (the amplitude is normalized to 1 for element 100). **e** RMS values for the last 2 ms of the calculated 100 ms of the displacements $x_j(t)$. **f** RMS value for the last 50 ms of the calculated 100 ms of the displacement $x_1(t)$, which is the generated SOAE, calculated for 11 values of the natural frequency of oscillator 1. The dashed line marks the element indicated with an arrow in panel **e**

In the next step of our analysis, things were left the same as for Fig. 4 except that the value of the damping parameter $\gamma _{0,100}$ was changed. In the absence of fluid coupling (α=0), element 100 changes from a damped oscillator into a self-sustaining oscillator if $\gamma _{0,100}$ is larger than 1.06. Here it was given the value 1.1, and the results are shown in Fig. 5.

The first thing to be noticed is the similarity between panels a to e in Figs. 4 and 5. This is despite the fact that the SOAEs in the two situations are generated by two different mechanisms: a global system in Fig. 4—in which all the elements are passive when working alone but active when working cooperatively—and a system with one local oscillator in Fig. 5 (in which this element is, by itself, self-sustaining).

The essential difference between the two systems is illustrated in panel f. In the global system, the SOAE at 3.14 kHz disappears if the natural frequency of oscillator 1 is close to this frequency. In the local system, on the other hand, the SOAE is strongest for this condition. An additional—and also crucial—difference is dealt with in the Discussion.

## A Closer Look at the Resonant Cavity

**Fig. 6:**
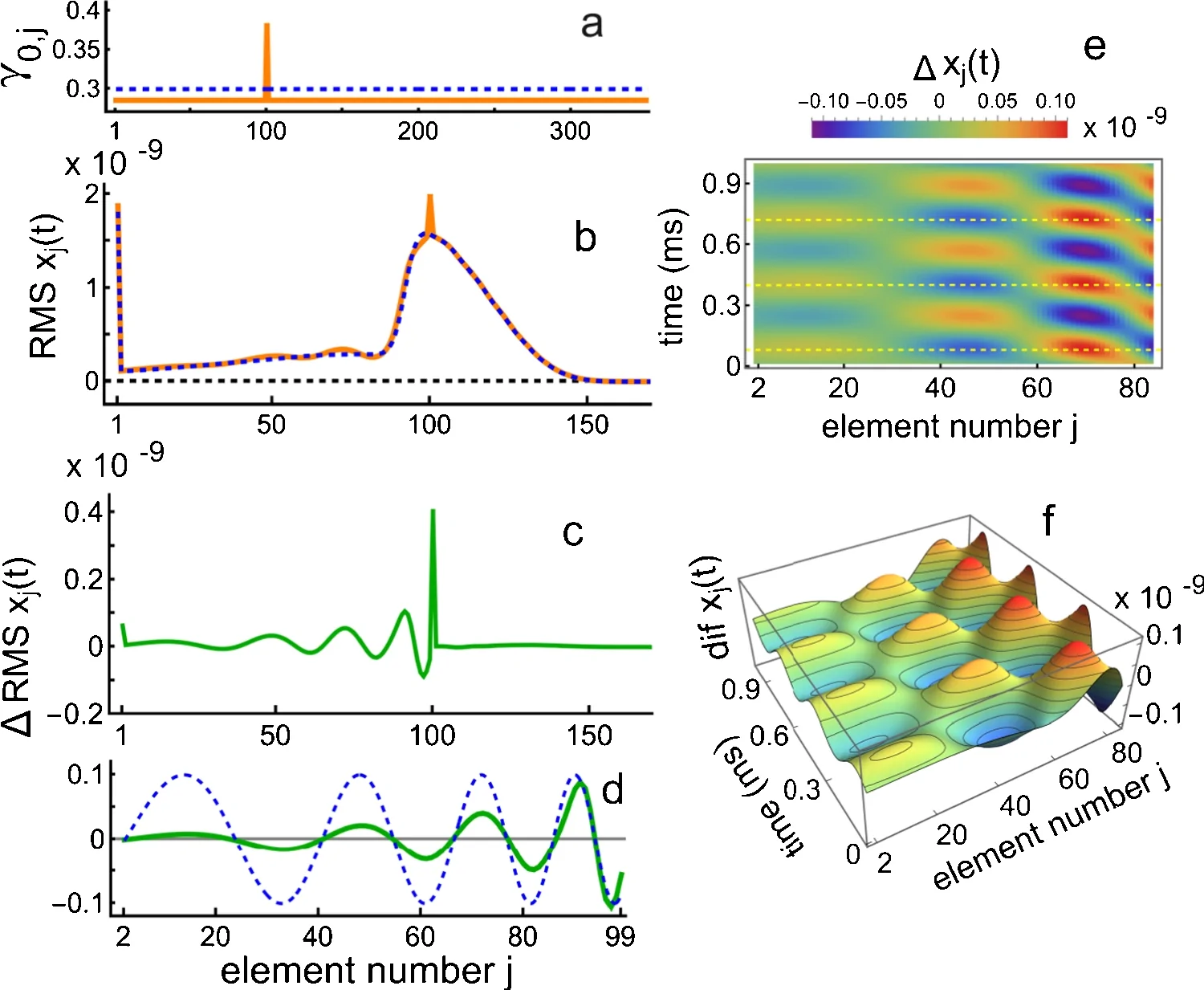
A standing wave in a resonant cavity emerges after considering two different damping profiles. In both cases, oscillator 1 (the middle ear) is driven by a sine wave of 3.12 kHz, but in one case, there is a single irregularity at element 100 in the array of damping parameters. **a** The array of damping parameters for the two situations. **b** Dashed blue line: RMS values without irregularity. Orange line: RMS values with irregularity. **c** Difference between the two curves in panel b. **d** Expanded view of part of c. The blue dashed curve is a compressed sine wave fitted to the zero-crossings of the green curve. See text for details. **e** Density plot for the standing wave, being the difference for displacements $x_j(t)$, for the situations with irregularity and without irregularities, for elements 2–85, during 1 ms. **f** Same as **e**, but now in a 3D-plot

To elucidate the system properties required to produce a standing wave within a resonant cavity, the previous calculations that gave the green curve in Fig. 3d (for $\gamma _0=0.3$), were repeated using the same set of parameters. This gave the dashed blue curve for the RMS values of the elements in Fig. 6b below. The calculation was then repeated after replacing the array of damping parameters $\gamma _{0,j}$ by the orange curve in Fig. 6a. This curve has a single irregularity at element 100, which is 0.1 larger than all the other damping parameters. The value for the other parameters (actually 0.2856) was found by trial and error to give the same RMS values for j>100, as for the situation without the irregularity. The result is the orange curve in Fig. 6b. The difference between the two curves (orange minus blue) in Fig. 6b is given in Fig. 6c.

The inference to be made from Fig. 6 is that the irregularity at element 100 is the low-frequency edge of a resonant cavity set up between oscillator 1 and element 100, and in which a standing wave is created.

The standing wave in Fig. 6 is the sum of a wave travelling in the direction of the irregularity with exponentially decreasing phase speed, and of a reflected wave travelling back with increasing speed (hence the nonuniform spacing of the zero-crossings). Empirically, the compressed sine wave through the zero-crossings in Fig. 6d was found to follow the equation $\Delta RMS x_j(t)=\alpha \sin [2\pi (\beta e^{\gamma j}-\delta )]$, with α=0.1, β=1.539, γ=0.01272, δ=1.581 (see Appendix 2 in [19] for details).

## An Irregular Array of Damping Parameters

The calculations to obtain Figs. 4 and 5 were repeated, except that the damping parameters were now made irregular so as to simulate cochlear roughness, which is a prerequisite for the generation of evoked otoacoustic emissions, according to Zweig and Shera [13]. Parameter $\gamma _0$, that controls the damping, was made randomly irregular for j=2 to *n*, so that $\gamma _{0,j}= \gamma _c+\delta _{sd}\chi _j$ (where $\chi _j$ is a normal distribution with mean 0 and standard deviation 1). This equation is not unlike equation (5) in [20], where cochlear roughness is introduced by perturbing basilar membrane stiffness. Following generation of the array, it was rotated to make element 100 the one with a large $\gamma _0$-value (0.522), as in Figs. 4 and 5.

**Fig. 7:**
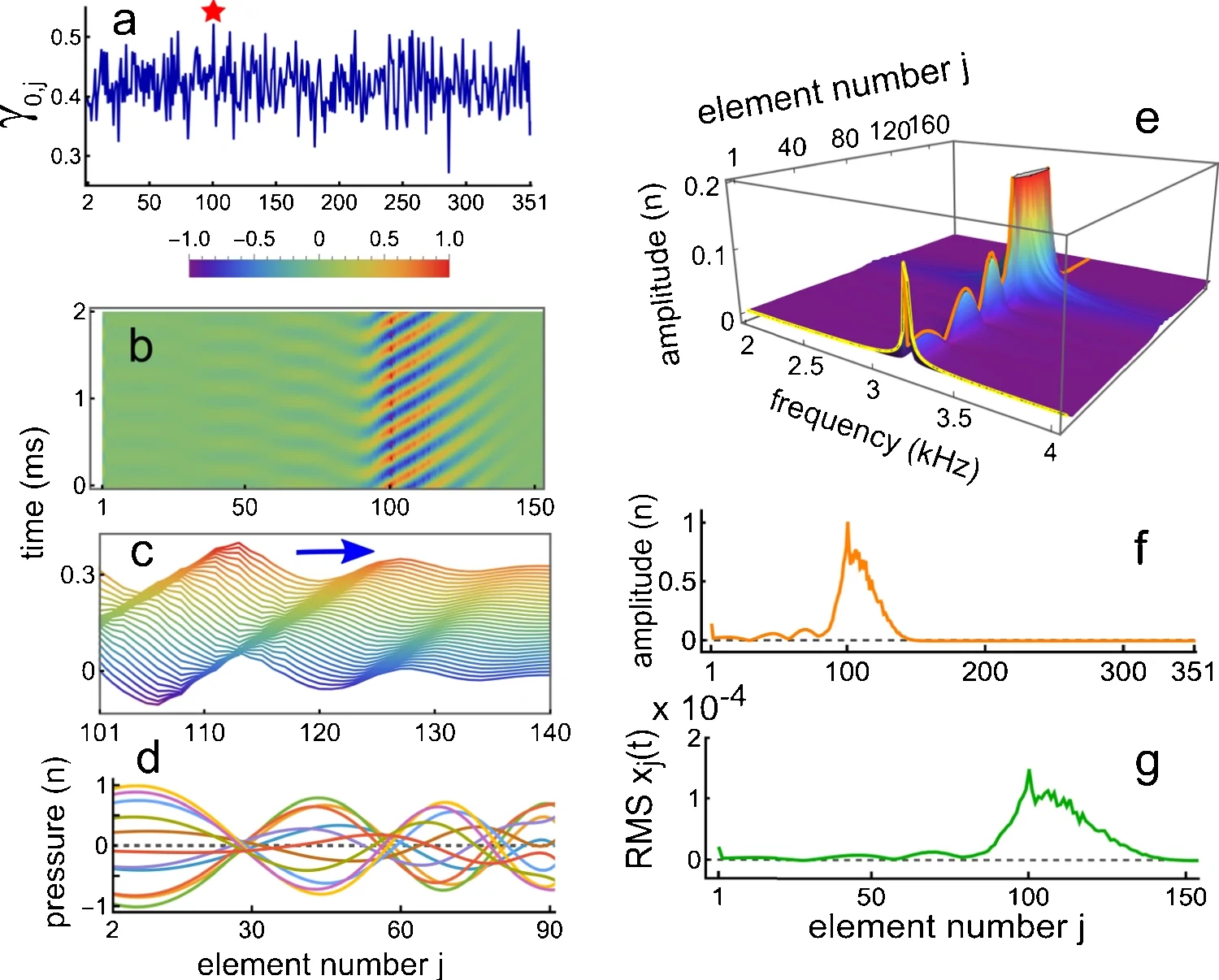
Self-sustaining oscillations in an array of the micro-mechanical elements, when their damping parameters $\gamma _{0,j}$ are randomly varied around 0.42 so as to simulate cochlear roughness. **a** The array of irregularities. The star marks the irregularity at element 100, with $\gamma _{0,100} = 0.522$. **b** Density plot for the last 2 ms of the calculated 100 ms of the displacements $x_j(t)$, for j=1 to 150 **c** Successive displacement profiles for elements 101 to 140 during one period in a waterfall plot, showing a damped wave travelling in the direction of the arrow. **d** Successive fluid pressure profiles along the array of elements during the last calculated period of oscillation, showing a standing wave. **e** A 3D-plot of frequency spectra for the last 50 ms of the calculated 100 ms of the displacements $x_j(t)$, for oscillator 1 (yellow line at 3.18 kHz), and for elements 2 to 185. The spectra around element 100 are truncated to better show the nodes and antinodes of the standing wave. **f** The orange line in panel **e**, extended out to element 351. **g** RMS values for the last 2 ms of the calculated 100 ms of the displacements $x_j(t)$

Calculations were done for $\gamma _c=0.42$ and $\delta _{sd}=0.04$ (for $\gamma _c=0.41$ and $\delta _{sd}=0.03$ all elements underwent damped oscillations), and for natural frequency $f_1 = 1$ kHz. The results are given in Fig. 7. We see that oscillation of the stapes, represented by $x_1(t)$, is equivalent to an SOAE at 3.18 kHz.

**Fig. 8:**
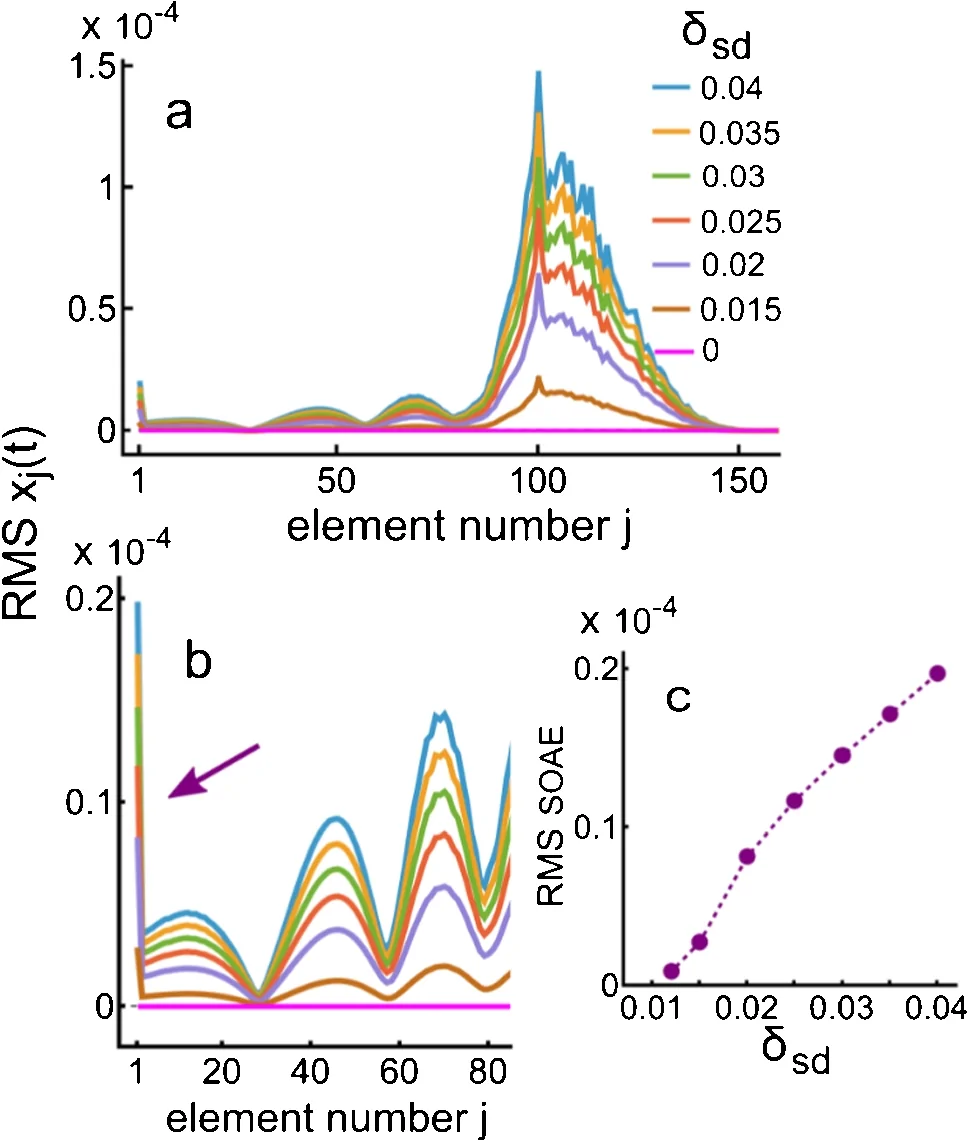
How different degrees of roughness affect the strength of SOAEs. Here, different standard deviations in the array’s damping parameters $\gamma _{0,j}$ were used to simulate roughness. **a** RMS values of the displacements $x_j(t)$ for the last 2 ms of a calculated 100 ms, for 7 different values of the standard deviation $\delta _{sd}$ in $\gamma _{0,j}$, but keeping $\gamma _{0,100}$ at 0.522. **b** Detail of a. The arrow marks RMS $x_1(t)$ (representing the strength of the generated SOAE). **c** RMS $x_1(t)$ for different values of $\delta _{sd}$. (The dot at $\delta _{sd} = 0.012$ has no corresponding curve in panels **a** and **b**)

The only notable differences between Fig. 7 (with roughness) and Figs. 4 and 5 are the patterns of small irregularities between elements 100 and approximately 130 in Fig. 7b, c, f, and g. These patterns largely correspond with the profile of the (irregular) array of damping parameters over the same interval (similar to Fig. 7a).

## Roughness Helps Energize the System

To investigate the effect of the strength of the irregularities on the generated SOAE, the same calculations, as for Fig. 7g, were repeated for different standard deviations $\delta _{sd}$ of the irregular $\gamma _{0,j}$-array, while $\gamma _c$ was kept at 0.42, except for $\gamma _{0,100}$, being 0.522 throughout. For each arrangement, the RMS profile along the array of elements was calculated. The results are shown in Fig. 8.

It can be concluded from Fig. 8 that the stronger the irregularities, the larger the amplitude of the self-sustaining oscillations of the elements, including the strength of the SOAE at oscillator 1. Furthermore, at least for this choice of parameters, irregularities are needed in order to evoke an SOAE. Without irregularities, there are no self-sustaining oscillations.

However, while the result is clear graphically, the mechanism behind it is not immediately apparent. How and why should roughness in a set of oscillators lead to amplification of the SOAE? The answer is by no means clear to us, and we discuss this point later.

## Creating Multiple SOAEs

To elucidate the role that damping parameters play in the generation of SOAEs, we illustrate what happens when undamping is increased at two more isolated positions in the array. Here, the calculations for the same irregular array of damping parameters used to obtain the results in Fig. 7 were repeated after making two changes: increasing the standard undamping term $\gamma _{0,60}$ from 0.436 to 0.52, and $\gamma _{0,124}$ from 0.493 to 0.57. This change resulted in the generation of three SOAEs at frequencies of 2.20, 3.18, and 5.48 kHz, rather than the previous single SOAE at 3.18 kHz.

**Fig. 9:**
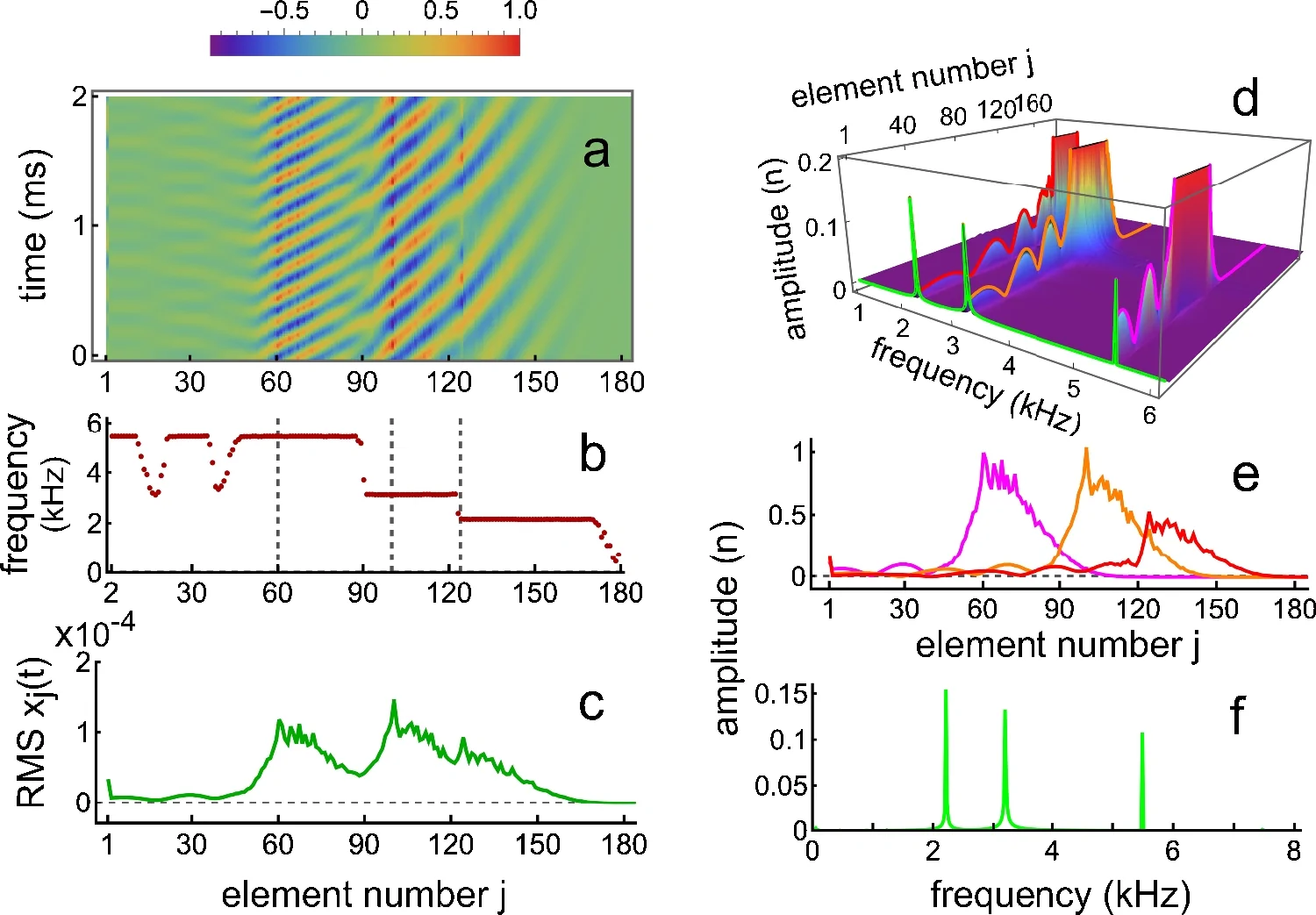
Self-sustaining oscillations in an irregular array of micro-mechanical elements when activity is increased by raising two undamping parameters (for elements 60 and 124) with respect to the situation for Fig. 7. **a** Density plot of displacements $x_j(t)$ for the last 2 ms of a calculated 100 ms, for j=1 to 180. **b** Frequencies of each of the individual elements revealing frequency clustering of neighboring elements into frequency plateaus. Frequencies were calculated from the average time between successive zero-crossings, for the last 10 ms of the calculated $x_j(t)$. The dashed vertical lines mark elements 60, 100, and 124. **c** RMS values for the last 2 ms of the calculated 100 ms of the displacements $x_j(t)$. **d** Frequency spectra for the last 50 ms of a calculated 100 ms of the displacements $x_j(t)$ in a 3D-plot, for oscillator 1 (green line, with peaks at 2.20, 3.18 and 5.48 kHz), together with spectra for elements 2 to 185. The spectral profiles are truncated to better show the nodes and antinodes of the standing waves. **e** The red, the orange, and the magenta lines in panel d without truncation. **f** Frequency spectrum for the three SOAEs generated at oscillator 1

The results are shown in Fig. 9.

After comparing Figs. 7 and 9, the main conclusion to be drawn is that Fig. 9 is effectively a three-way composite of the single arrangement in Fig. 7, leading to 3 resonant cavities instead of 1. Each cavity generates its own single-frequency SOAE, a process which Zweig [21] compared to how a laser creates a single-frequency (or wavelength) light beam.

## Spacing of SOAEs

To investigate the spacing of the peaks in frequency spectra of SOAEs, the calculations to obtain Fig. 9 were repeated for the same $\chi _j$ as for Fig. 7a, but now for somewhat higher values for the undamping parameters $\gamma _{0,j}$. This change led to more spectral peaks. Here $\gamma _c$ in $\gamma _{0,j}=\gamma _c+\delta _{sd}\chi _j$ was set at 0.5 and $\delta _{sd}$ at 0.05.

**Fig. 10:**
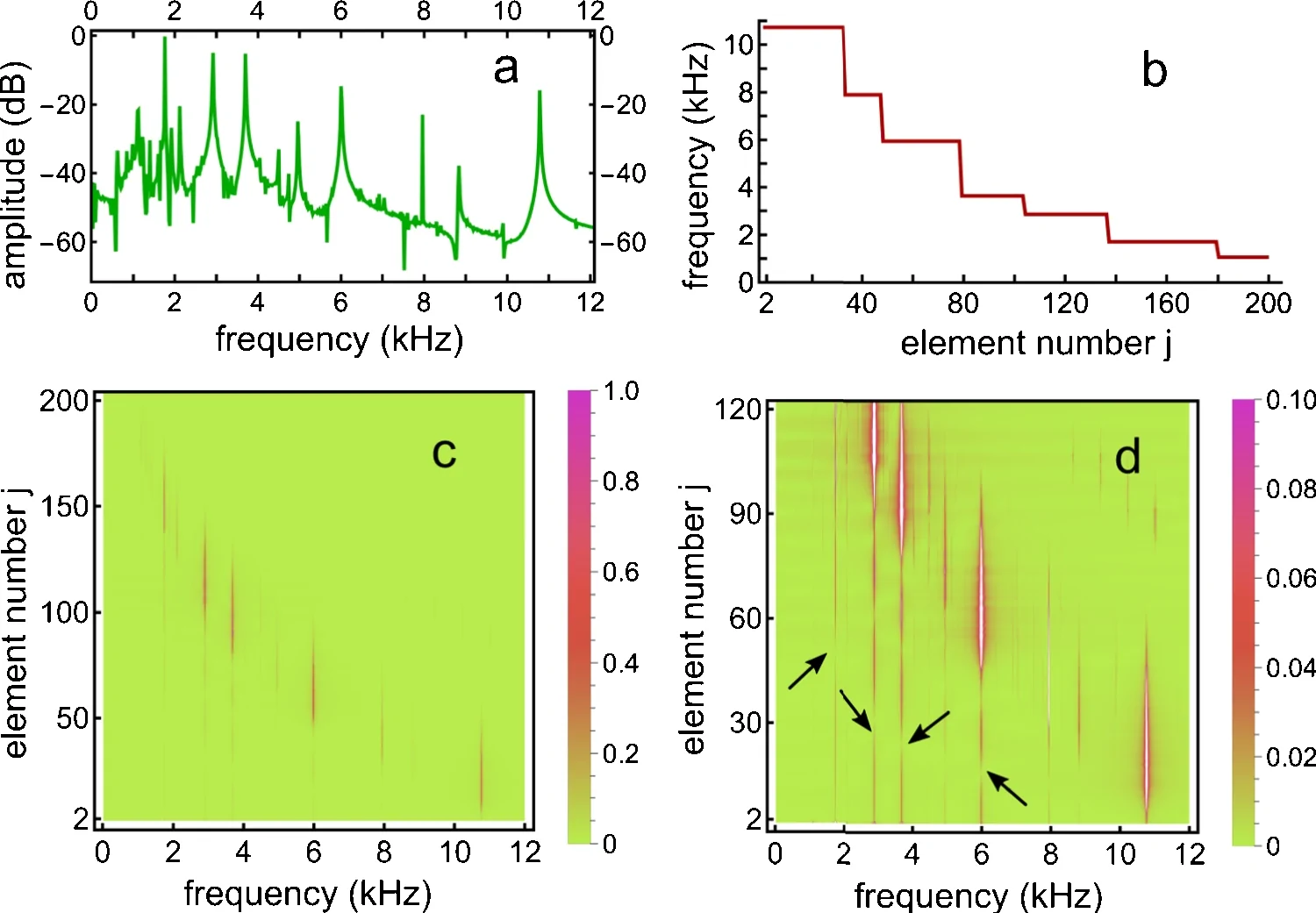
Multiple SOAEs can be generated by increasing the values of the undamping parameters in an irregular array of micro-mechanical elements. **a** Frequency spectrum for oscillator 1, showing the generated SOAEs. **b** Frequency with the largest amplitude in the spectrum of each of the individual elements, revealing that neighboring elements cluster into 7 discrete frequency plateaus. **c** Density plot of the frequency spectra of elements 2 to 200. **d** Expanded view of **c**. The arrows indicate standing wave nodes, which can also be seen in Figs. 4d, 5d, 7e, and 9d

The results are shown in Fig. 10.

**Fig. 11:**
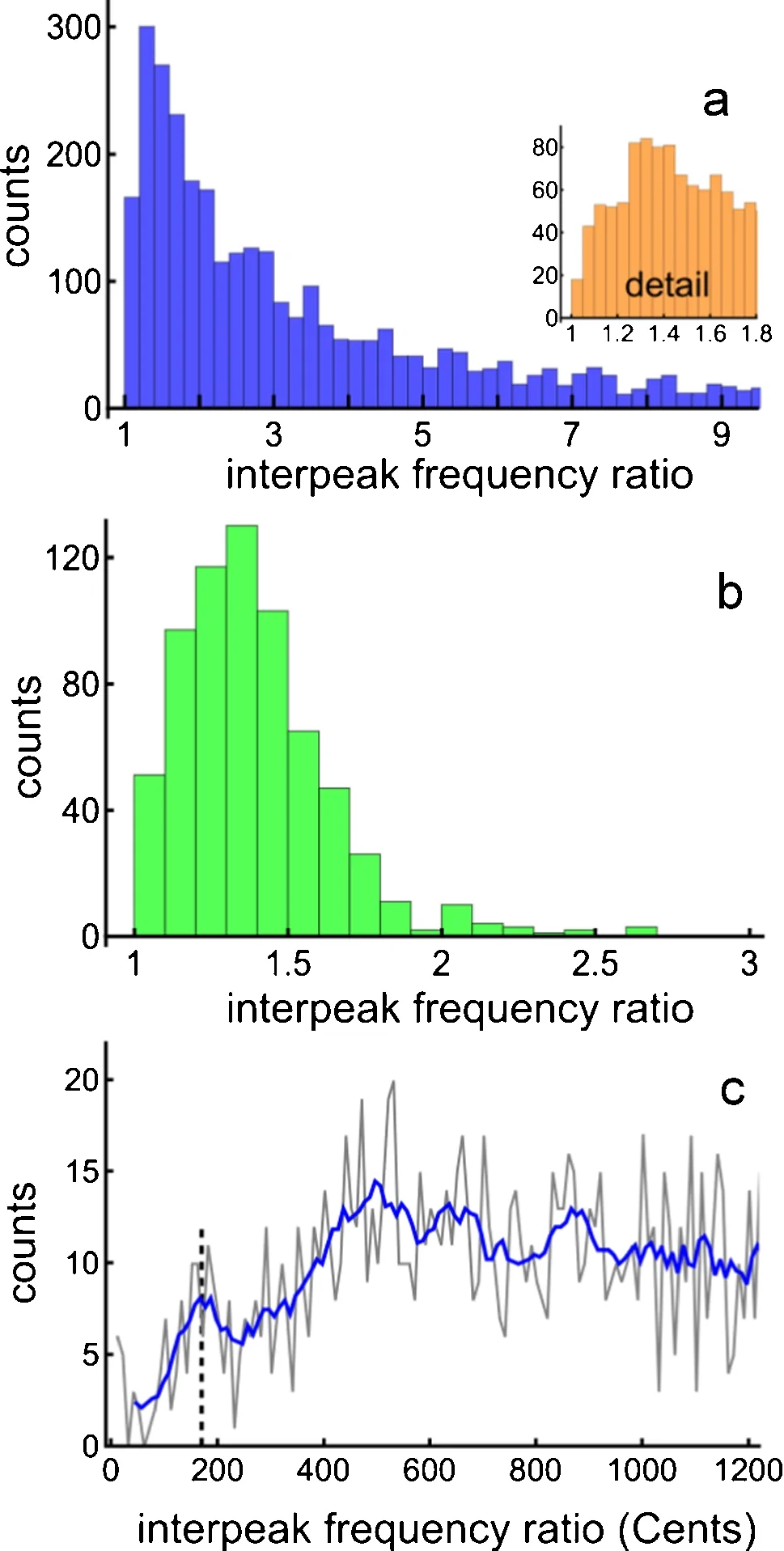
Distributions of interpeak frequency ratios in an SOAE spectrum, for 80 different spectra, with an average of 8.1 peaks per spectrum. **a**. Distribution of 2971 ratios between an SOAE frequency and all the lower frequencies in the spectrum. **b** Frequency ratios of neighboring peaks only. **c** Frequency ratios between 1 and 2 of panel a set out on a Cent scale (0 to 1200 Cents). Thin gray line: with a resolution of 10 Cents; blue line: with an 8-point moving average. The dashed vertical line is at 170 Cent, corresponding to a ratio of 1.10

A revealing feature of Fig. 10 is that the largest SOAE peaks in Fig. 10a correspond in frequency with those of the plateaus in Fig. 10b. There appears to be a certain regularity in the size of the steps, and to investigate this aspect, the interpeak frequency ratios were calculated by repeating 80 times those used to obtain Fig. 10b (each time with a newly generated $\chi _j$-array having a normal distribution with mean 0 and standard deviation 1). On average, there were 8 plateaus, and for each plateau, the ratio of its frequency with that of all the lower frequencies was calculated ($f_j/f_{j+1},f_j/f_{j+2},f_j/f_{j+3},...$). The distribution of all ratios obtained in this way is given in Fig. 11a. Also calculated were the ratios of neighboring plateaus only ($f_1/f_2,f_2/f_3,f_3/f_4,...$), with their distribution shown in Fig. 11b.

Of relevance, Bergevin et al. [22] investigated intrapeak and interpeak correlations in SOAE spectra of 8 humans, 8 barn owls, and 8 green anole lizards. They found that these spectra had, on average, 9.1, 11.8, and 10.9 peaks. For all three species, the interpeak frequency ratio distribution in Fig. 3B of [22] has a maximum at a ratio between 1.2 and 1.4 for all 3 species. The shape of their distribution for humans resembles Fig. 11a here, where it has a maximum at a ratio of 1.35.

Braun [23] investigated the frequency ratio of 5245 pairs of human SOAEs. Their distribution—both for females and for males—had a maximum at 100 Cent, corresponding with a ratio of 1.06. There were no peaks at multiples of 100 Cents (i.e., at 200, 300, 400). Figure 11c here should be compared with Fig. 1 in [23].

## Discussion

### Global Versus Local

If Figs. 4 and 5 are compared it can be seen that for both conditions—global and local—an SOAE is generated within a resonant cavity. For the global situation, a necessary condition is that wave reflection at the basal end of the cavity needs to be sufficiently strong to maintain the self-sustaining oscillation.

For the global case, however, if $\gamma _{0,j}$ is given a value smaller than 0.32, it can never produce any self-sustaining oscillation—all the oscillators are damped. As Shera [3] explains: “the round-trip gain no longer matches the losses due to internal damping and emission into the ear canal.” Or, in terms of our model, the undamping by feedback pressure $P_a$ in the Neely and Kim micro-mechanical element (Fig. 16 in Appendix 1) is too small.

**Fig. 12:**
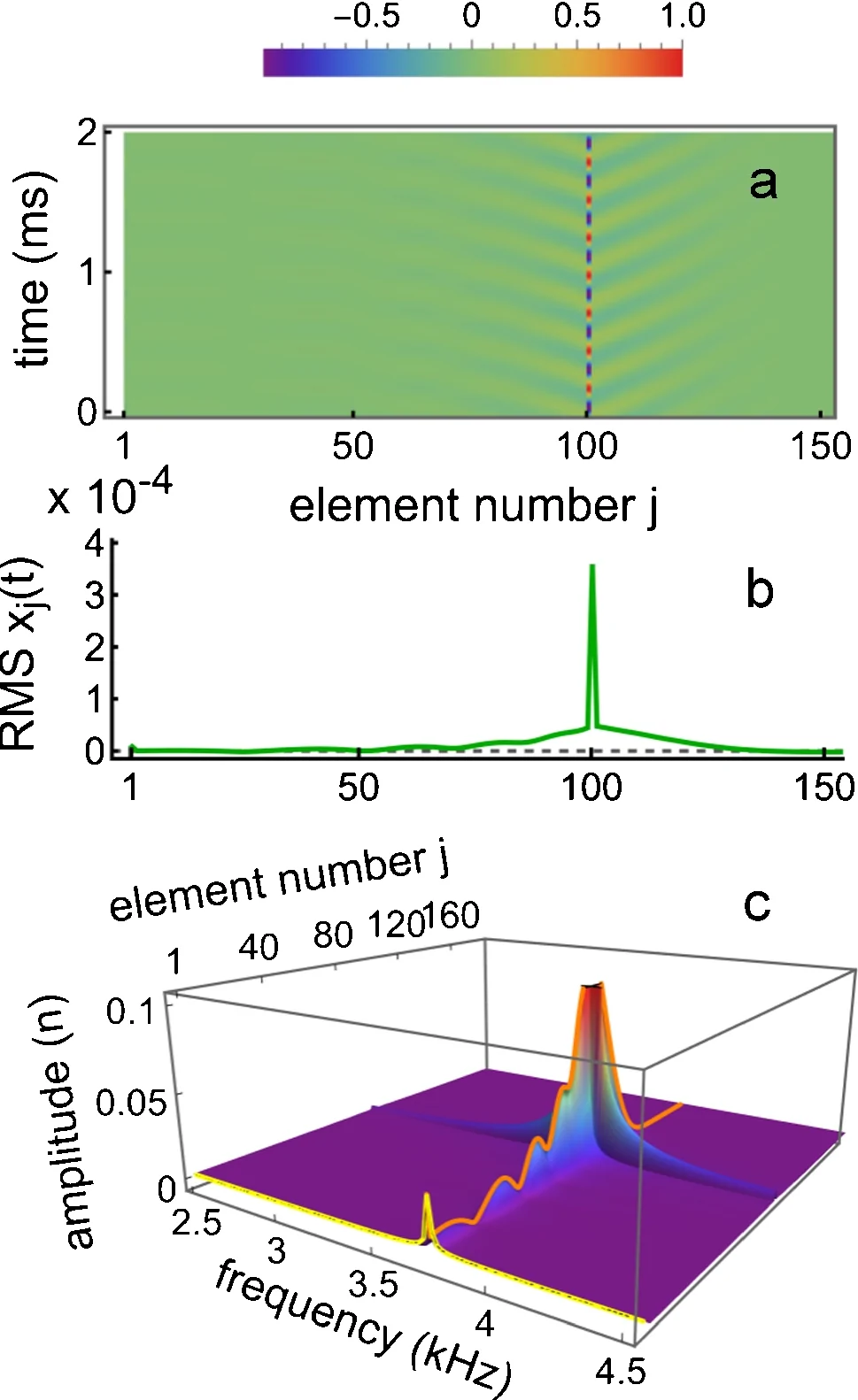
Self-sustaining oscillations in an array of the micro-mechanical elements, where the damping parameters contain a single irregularity, that gives rise to a single self-sustaining oscillator. **a** Density plot for the last 2 ms of the calculated 100 ms of the displacements $x_j(t)$, for j=1 to 150. **b** RMS values for the last 2 ms of the calculated 100 ms of the displacements $x_j(t)$. **c** A 3D-plot of frequency spectra for the last 50 ms of the calculated 100 ms of the displacements $x_j(t)$, for oscillator 1 (yellow line at 3.72 kHz), as well as for elements 2 to 185. The spectra around element 100 are truncated to better show the nodes and antinodes of the standing wave

Quite different is the situation for the single self-sustaining local oscillator: even for all $\gamma _{0,j}$ being zero, except for element 100 ($\gamma _{0,100}$ remains at 1.1), a self-sustaining oscillation is generated, although it has a much smaller SOAE amplitude and smaller standing wave in the resonant cavity. This can be verified by comparing the 3D-plots in Figs. 5 and 12.

It can be concluded that in the global situation, the creation of a resonant cavity is a necessary condition for the generation of an SOAE. On the other hand, with a local oscillator, the resonant cavity is merely a by-product.

### Cochlear Standing Waves in Resonant Cavities

More than 30 years ago, Wit et al. [24] introduced wavelet analysis as a tool for time-frequency analysis of click-evoked otoacoustic emissions from human ears. They found that the introduction of a slight irregularity in the frequency-to-place map of the inner ear gave rise to outcomes that looked remarkably similar to those obtained from actual ears. They also showed that adding periodicity to the frequency-to-place map to emulate Bragg reflection, as proposed by Strube [25] to explain the relation between time delay and frequency in click-evoked otoacoustic emissions, gave unrealistic results.

In related work, Wit et al. [26] showed how the presence of small irregularities in the array of central frequencies of the individual filters in a bank of linear bandpass filters leads, in a rather straightforward way, to(quasi) periodicity in (synthesized) spectra of click-evoked otoacoustic emissions.

One year earlier, Shera and Zweig [12] expanded on Strube’s [25] “cochlear washboard” proposal to explain periodicity in the spectrum of evoked otoacoustic emissions in terms of coherent scattering off irregularities, creating a backward-travelling wave that interferes with the stimulus tone.

**Fig. 13:**
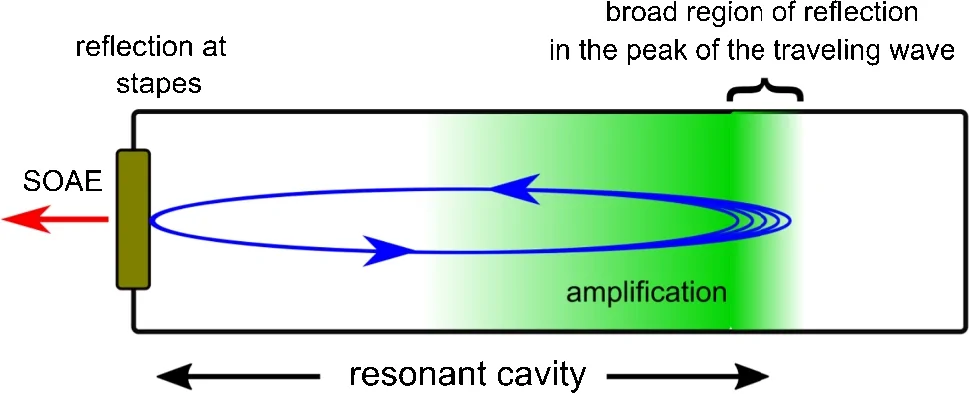
The cochlea acting as a resonant cavity. Blue arrows: forward and backward travelling waves in the inner ear, creating a self-sustaining standing wave. Green shading: region of amplification of the standing wave. Note the multiple reflection points in the region of reflection

Two years later, Zweig and Shera [13] elaborated this model and showed that “coherent reflection filtering” can also generate SOAEs and explain periodicity in their spectra. Essential in the coherent reflection filtering paradigm is, in contrast with Kemp’s [27] original standing wave model, where the backward travelling wave originates from a point reflection, that this wave “necessarily arises over a region equivalent to the span of many hair cells.” This is illustrated in Fig. 13, which is a somewhat modified version of Fig. 8 in [14].

Applied to the present model, this means that reflection needs to occur at more than a single micro-mechanical element. In this way, reflected waves that are in phase amplify each other ( hence the term “coherent reflection”).

Shera and Zweig [13] describe coherent reflection as follows: “...wave reflection is largely localized to a relatively broad region containing many reflectors, which are arrayed, in general, at a superposition of many spatial frequencies. Although each of the many reflected wavelets originates from a different location within the peak region, wavelets reflected by a particular spatial frequency, denoted $\hat{K}$, undergo a phase change due to scattering by the irregularity that exactly compensates for the phase change due to wave propagation forth and back to the point of reflection. Such wavelets therefore reflect coherently, and their sum dominates the net backward-traveling wave.”

Now, how does this situation apply to the global/local distinction we have made?

In the case of a local oscillator, Fig. 5 shows that when an SOAE is generated by a local oscillator, its source is a single element in the array of micro-mechanical elements. This single element bounds a resonant cavity—in which the standing wave with the frequency of the SOAE is generated—at its apical (low frequency) end. And although amplification (the green region in Fig. 13) generates a stronger SOAE, it is not needed, as is shown in Fig. 12.

A similar situation applies in the global case (Fig. 4). In this case, reflection occurs at a single element (*j*=100), which is at the apical end of a resonant cavity. There is no region of reflection spanning more than one element. And the wave propagating in the apical direction starts immediately at element 101, next to the apical border of the resonant cavity (see Figs. 4b and 7c). With the local case, an essential difference is that sufficient amplification (undamping) and reflection at the stapes are needed in order to generate an SOAE.

**Fig. 14:**
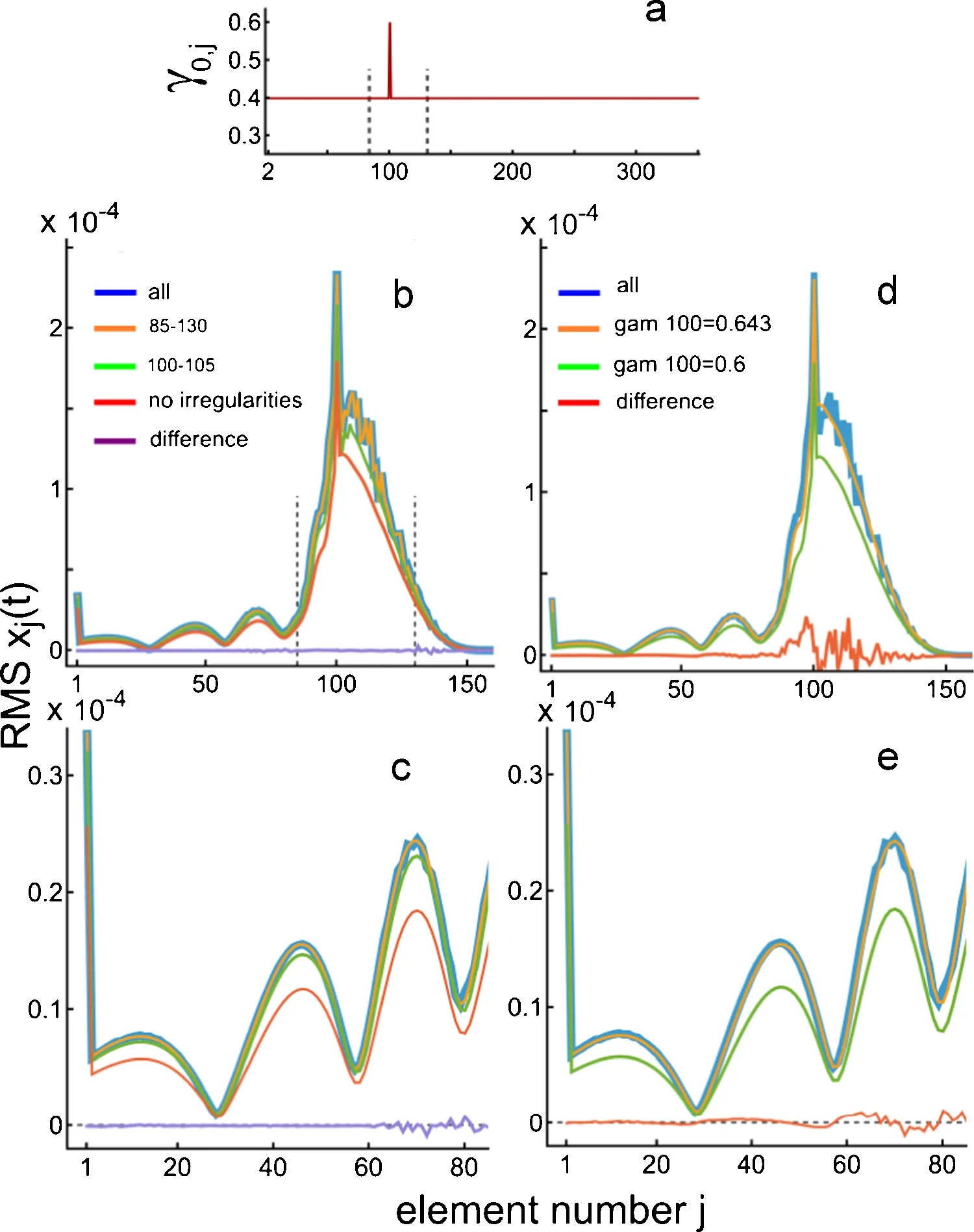
Where do irregularities matter the most? **a** Array of $\gamma _{0,j}$ values with no irregularities (except for $\gamma _{0,100} = 0.6$). The dashed lines mark elements 85 and 130. **b** RMS values for the elements for five situations.“all”: irregularities all along the array. Same array as in Fig. 7a, but with $\gamma _0=0.4$ and $\delta _{sd}=0.04$. “85–130”: irregularities from j=80 to j=130. “100–105”: irregularities from j=100 to j=105. “no irregularities”: situation as in panel a. “difference”: difference between the blue and the green line. **c** Detail of b. **d** RMS values for the elements for 4 situations.“gam 100=0.643”: no irregularities, except for $\gamma _{0,100}=0.643$. “gam 100=0.6”: no irregularities, except for $\gamma _{0,100}=0.6$ (as the red line in b). “difference”: difference between the blue and the orange line. **e** Detail of d

It is clear from Fig. 8 that increasing the strength of the irregularities in the array of undamping parameters $\gamma _{0,j}$ amplifies the standing wave and the SOAE (Fig. 8b and c). By setting $\delta _{sd}$ at zero for different regions of the array of irregularities, we found by trial and error that if irregularities are restricted to the region from element 85 to element 130, it amplifies exactly as much as when irregularities are present all along the array. In other words, irregularities for j=2-84 and j=131-351 play no role. But having irregularities only for elements 100–105 is enough to amplify the displacements of the elements, as can be seen in Fig. 14b and c.

Instead of introducing irregularities, the displacements can also be made larger by increasing the value of $\gamma _{0,100}$. In Fig. 14d and e, it can be seen that increasing $\gamma _{0,100}$ from 0.6 to 0.643 gives exactly the same RMS value as for the situation with irregularities present all along the array.

**Fig. 15:**
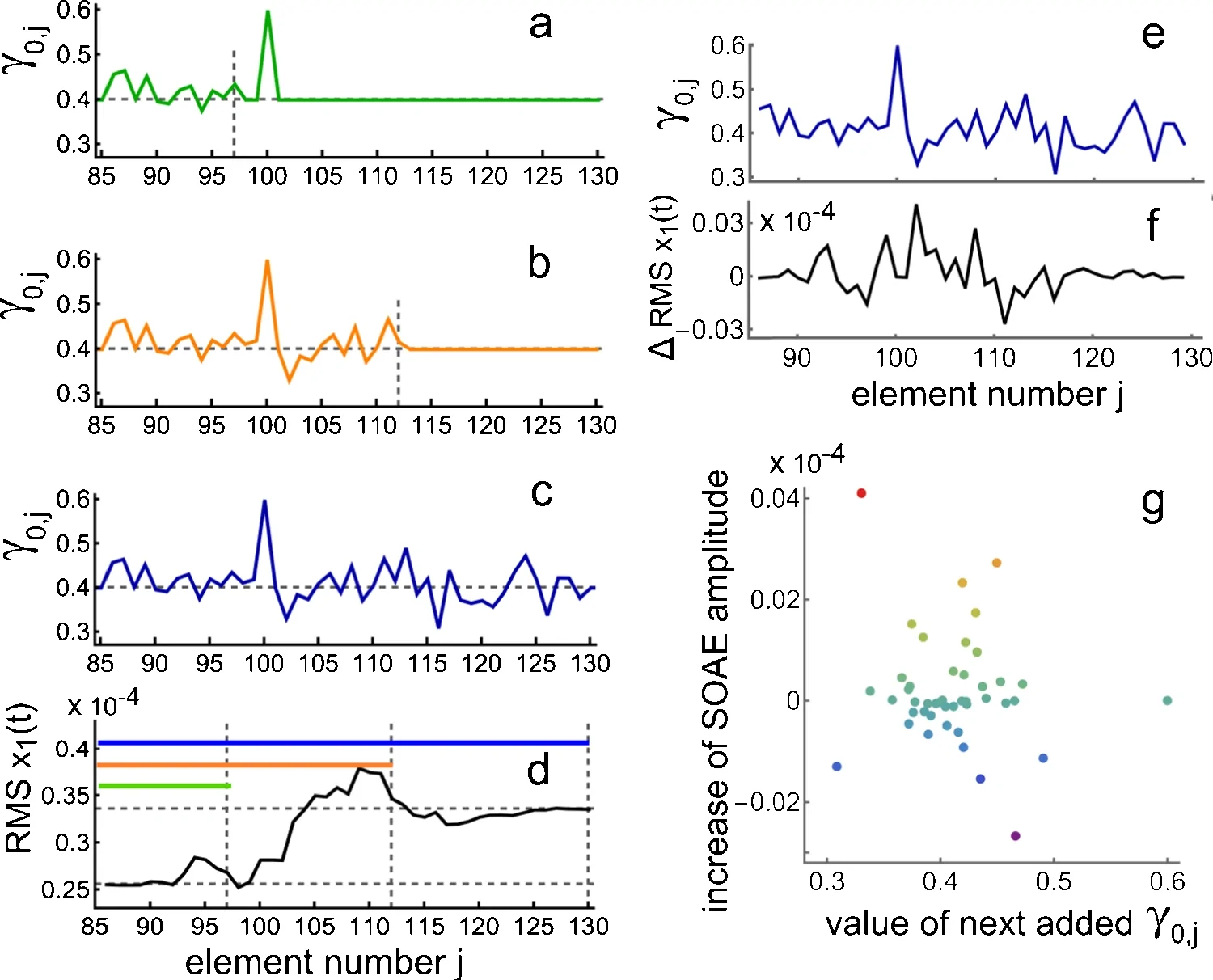
Where irregularities matter: the result of progressively increasing the extent of irregularities. **a** Irregularities for elements 86–97 (marked with the green line in d). All other $\gamma _{0,j}$ are 0.4 (except for element 100). **b** Irregularities for elements 86–112 (marked with the orange line in d). **c** Irregularities for elements 86–130 (marked with the orange line in d). **d** SOAE amplitude increasing from its value for no irregularities at all (lower horizontal dashed line) to the value for irregularities all along the array (upper horizontal dashed line). The colored lines mark the number of included elements. **e** As panel c. **f** Increase of the SOAE amplitude (as shown in panel **d**) if one more irregularity $\gamma _{0,j}$ is added. **g** Increase in SOAE amplitude (shown in panel **f**) plotted against the value of the next added irregularity (shown in panel **e**)

**Fig. 16:**
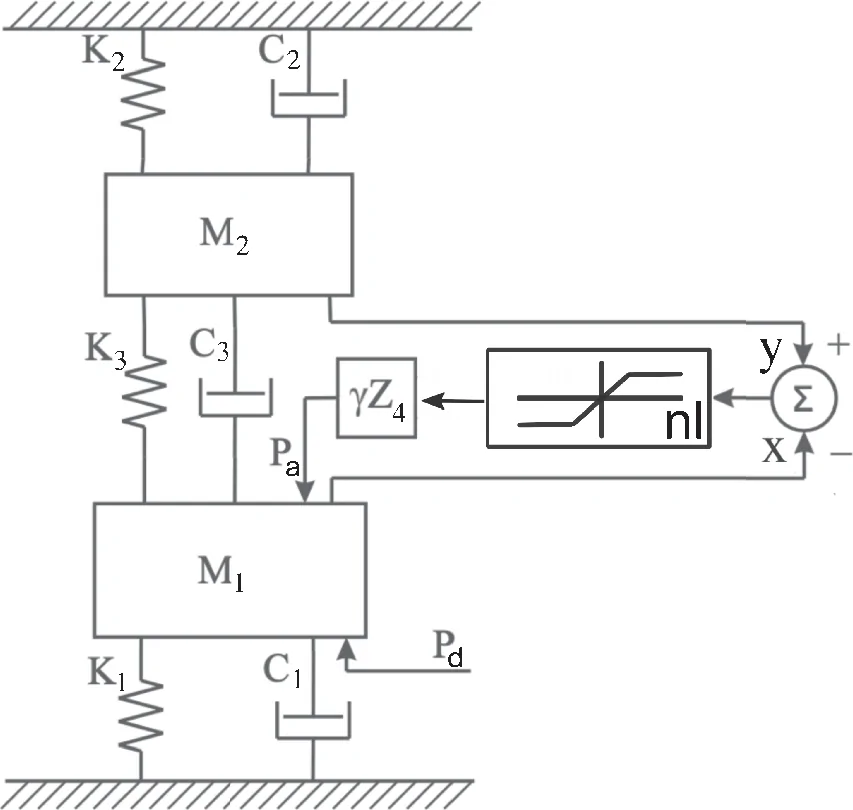
Adapted version of Neely and Kim’s micro-mechanical element. Note the undamping parameter γ (as in $\gamma Z_4$), in which larger γ means less damping (or more undamping)

It is shown in Fig. 14b and c that irregularities from element 85 to element 130 create the same amplitude for the standing wave and the SOAE as irregularities all along the array. To investigate this in more detail, the RMS value of oscillator 1 (the SOAE) was calculated step-by-step, starting at element 86 and including progressively more irregular elements. The result, as shown in Fig. 15d, is that the level of the SOAE increases from a value for no irregularities all along the array to a value for irregularities all along the array.

Figure 15d shows a more or less gradual increase for the amplitude of the generated SOAE if the number of irregular elements increases from 12 (at element 97) to 45 (at element 130). By adding one more irregular element, we see no systematic increase in amplitude at regular intervals, which we would expect if the phase of the wavelet reflected by this element positively contributed to the amplitude of the standing wave. Furthermore, it can be concluded from Fig. 15 e, f, and g that there is no correlation between the profiles for the array of irregularities and the increase of SOAE amplitudes.

In this context, it is worth looking again at Fig. 14b and c, which show that more irregularities mean stronger reflection, not more amplification. If it were a case of amplification, then one would expect a contribution of the irregularities in the region between elements 2 to 85, where the standing wave is created. Yet we see that only 5 irregularities (elements 101–105) contribute almost as much to the amplitudes of the standing wave and the SOAE as all the other irregularities.

Furthermore, Fig. 15d shows that the irregularities contribute in a complex way. In some regions (for instance, 110–115), increasing the number of contributing irregularities even decreases the amplitude of the SOAE.

Finally, the coherent reflection explanation calls for reflections from a particular spatial frequency: a structure that is periodic across position in space. Such behavior is also not seen in Fig. 15.

In the end, it must be concluded that the time-domain model, as presented here, gives a clear indication that reflection at one or more irregularities is essential for the generation of spontaneous otoacoustic emissions. But how the reflected signals add up to create a sufficiently strong standing wave is not immediately clear.

Ku et al. [28], applying the same time-domain state-space model [16] as we do here, observed “the salient features of Zweig and Shera’s theory.” Among other things, they calculated the statistics of unstable frequencies (self-sustaining oscillations) for random spatially dense variations in gain all along the array of micro-mechanical elements. They found that the average spacing of adjacent unstable frequencies agreed with the preferred minimum distance in human SOAE data. They gave no information about how reflections from individual irregularities could add up to contribute to the amplitude of a self-sustaining oscillation.

Since Ku et al. [28] do not give any information about how reflections from individual irregularities add up to contribute to the amplitude of a self-sustaining oscillation (and we could not find other published time-domain models that investigate the generation of SOAEs by reflection from many irregularities) we do not really know what might be convincing evidence of the presence of coherent reflection, at least as intended by Zweig and Shera [13].

### Frequency Plateaus

Figure 9 shows that the three peaks in panel f are generated in three partly overlapping resonant cavities. Also in Fig. 10, standing waves, generated in separate resonant cavities, can be seen. Each of these waves corresponds to an SOAE-peak in panel a. Similarly, clear distinct standing wave patterns—and corresponding frequency plateaus—can also be seen in Fig. 3 of Epp et al. [29], where the generation of SOAEs is also modelled with a one-dimensional array of micro-mechanical elements. However, in this case, while the elements have velocity-dependent damping and a velocity-dependent feedback stiffness term [30], they are different from the Neely and Kim element.

Frequency plateaus are also generated in the model by Fruth et al.[17] for the mammalian ear (see their Fig. 6). In this one-dimensional model, the 3500 (!) micro-mechanical elements are Hopf oscillators that are coupled hydrodynamically and via elastic and dissipative elements. The latter type of coupling is the same as in the model for the lizard ear by Vilfan and Duke [4]. In the light of the present work, it appears relevant that Fruth et al. [17] find that SOAEs in their model “are elicited by synchronization of local oscillators, which is in agreement with the SOAE generation mechanism found in other vertebrates. This is in contrast to the notion of SOAEs arising of a global phenomenon from wavelike excitations.”

The conclusion from the different approaches seems to be that frequency plateaus are a universal phenomenon underlying the formation of SOAEs, independent of the model used.

### Spacing Order

Spectra with multiple SOAEs from a human ear show spacing order [31]) and a preferred minimum distance (PMD) on a logarithmic frequency scale. Braun [23] determined the PMD between spectrally neighboring SOAEs to be 1 semitone (100 Cent), or a frequency ratio Δf=1.06 ($\Delta f = 2^{(PMD/1200)}$. It can be concluded from Braun’s Fig. 1 that there is no perfect order: the distributions in this figure have a long, gradually decaying tail, without local maxima at multiples of the PMD. Shera [14] found the same value for the PMD with his Fig. 2 having a maximum at $N_{SOAE}=17$. Using the relation $\Delta f=(2N_{SOAE}+1)/(2N_{SOAE}-1)$ this also yields a preferred minimum ratio Δf of 1.06.

A frequency ratio of 100 Cent corresponds with 1 semitone in musical terms. This prompted Bell and Jedrzejczak [32] to suggest that “the inner ear may play a role in constructing a natural theory of music.”

Order in the spectra with multiple SOAEs in the present work was investigated by repeatedly calculating SOAE spectra, each time for a newly generated irregular array of damping parameters. Just like for spectra of human SOAEs, Fig. 11a and b reveal a PMD, but at a frequency ratio of 1.35 (or 520 Cents). This corresponds well with the positions of the maxima in the distributions for human, owl, and anolis in Fig. 3B of [22]. But not with the distributions given by Braun [23] or Shera [14] (although Fig. 11c shows a small local maximum at a ratio 1.10, not 1.06).

More light can possibly be thrown on the discrepancy between our results and those of Braun [23] and Shera [14] by repeating the model calculations with other sets of parameters. For instance, a larger standard deviation of the array of damping parameters generates more spectral peaks. What remains to be explained is the difference between Braun’s and Shera’s distributions and that of Bergevin et al.

### Non-mammalian Ears

The question naturally arises as to whether the global oscillator paradigm is restricted to just mammalian ears, since it is generally considered that, for the lizard ear at least, a local oscillator model is appropriate. Traveling waves and resonant cavities do not seem to be present in non-mammalian ears.

Thus, when Vilfan and Duke [4] and Gelfand et al. [5] modelled the generation of SOAEs in the ears of lizards, they constructed an array of coupled active elements (in the first case, 80 Hopf oscillators and in the second, 110 Van der Pol oscillators). In both models, the SOAEs are simply peaks in the power spectrum of the oscillator displacements.

Slightly differently, in the model of the lizard ear by Wit and Bell [7], all the inner ear oscillators were active Hopf oscillators, and these were considered to directly drive the basilar papilla. The motion of the papilla was then coupled to the tympanic membrane by incompressible inner ear fluid, which in turn created SOAEs.

Moreover, even the most elementary ears of frogs emit SOAEs (for a review, see Van Dijk et al. [33]). Their most likely source is the amphibian papilla: an arrangement of hair cells, covered with tectorial membrane, located on the dorsal wall (top) of the amphibian recess. However, unlike for lizards, there is no comparable model for the generation of SOAEs in frogs, although Bell [34] sets out a possible “local oscillator” mechanism: oscillations of hair cells are directly transported to the tympanic membrane by fast pressure waves in the inner ear fluids.

### Other Irregularities

All the results presented above are for irregularities in the array of damping parameters $\gamma _{0,j}$. But this choice is not the only one able to generate SOAEs. Self-sustaining oscillations can, in fact, also be generated by other sorts of irregularities. One such example based on irregularities in stiffness is given in Appendix 2.

## Conclusions

By modelling the cochlea as a graded array of micro-mechanical elements—two feedback-coupled masses with variable degrees of counteraction of their damping—our numerical computations provide a number of insights into how spontaneous emissions might arise. The model replicates a number of SOAE properties.

One notable aspect is the way it may help resolve the question of whether SOAEs are of local or global origin, suggesting that the distinction is really a matter of how the irregular array of damping parameters is arranged.

Another point of interest is the appearance in the model of a standing wave within a resonant cavity. All elements within the cavity oscillate at the same frequency, which is the frequency of the generated SOAE.

Our time-domain model is also able to simulate roughness, and although the results may be indicative, the interpretation is not clear-cut. The calculations show that increased roughness increases the size of SOAEs, but our model is limited in explaining how such an amplification arises.

In summary, the present model shows a number of interesting features that help explain some aspects of cochlear mechanics, but at the same time, it also gives rise to a number of unanswered questions which need explanation by further exploration.

## Figures and Tables

**Fig. 17:**
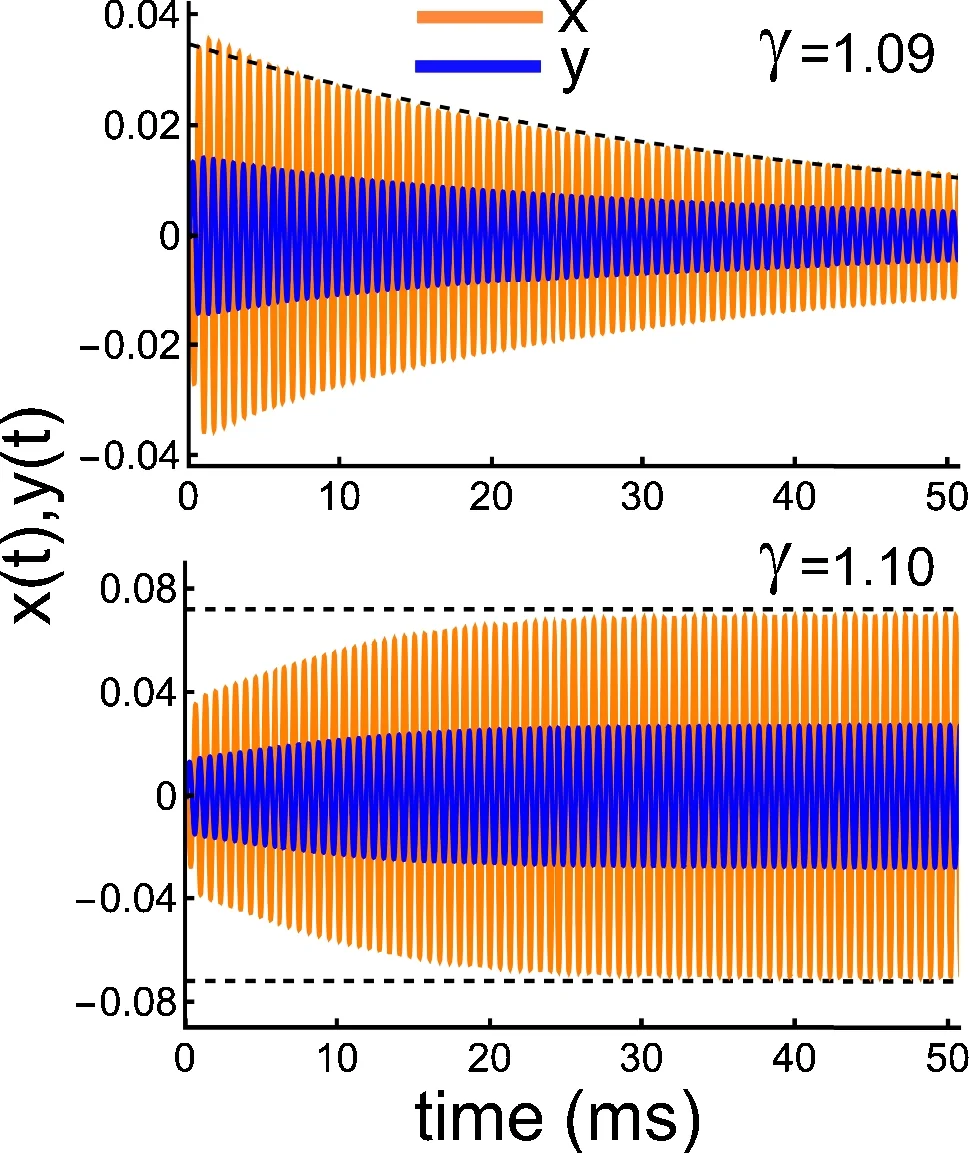
Responses *x*(*t*) and *y*(*t*) for the two coupled oscillators in Fig. 16, for two values of damping parameter γ. The dashed line in the upper panel is a fit to the envelope of the orange curve with $x=\alpha e^{\beta t}$

**Fig. 18:**
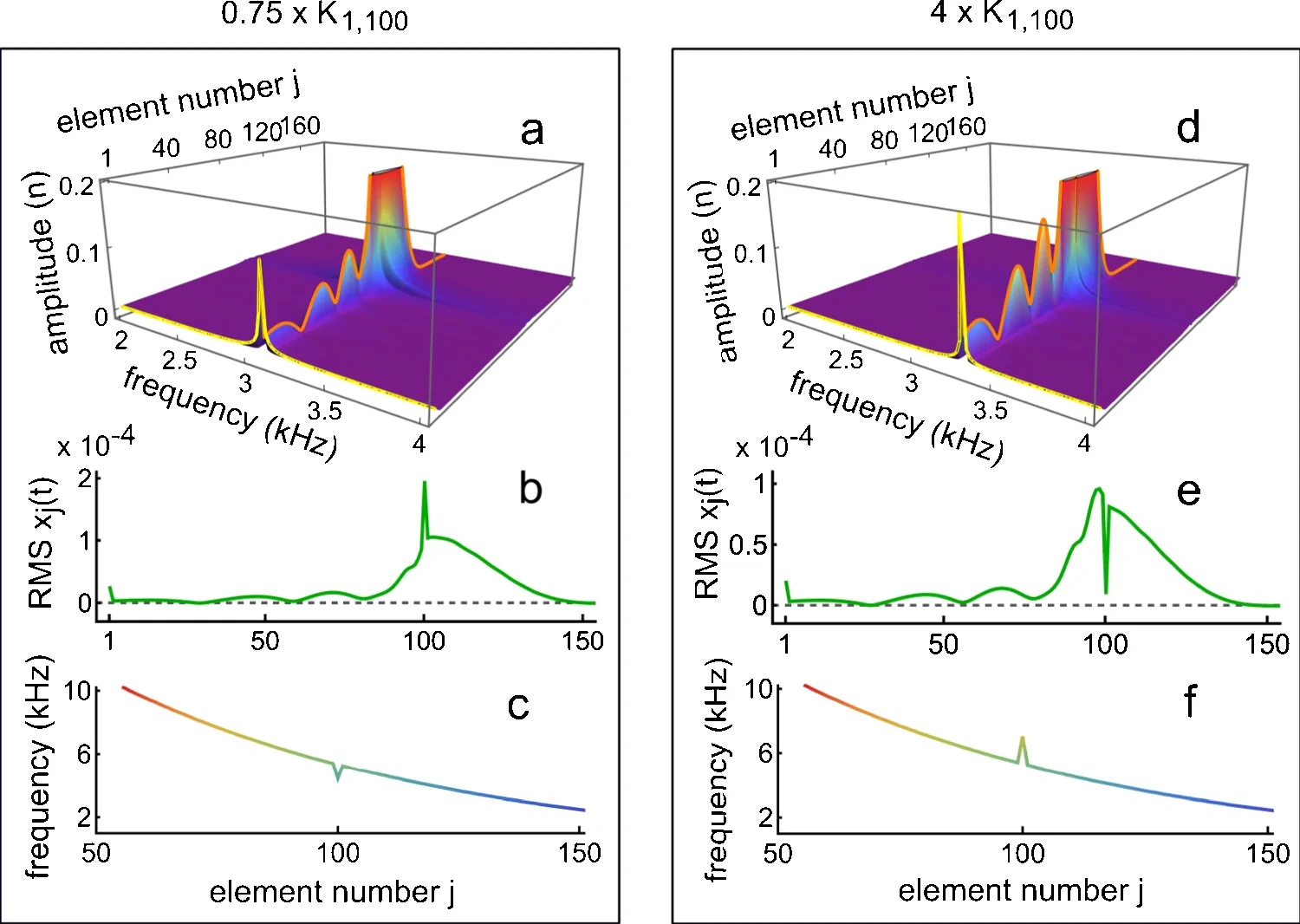
Self-sustaining oscillations in an array of micro-mechanical elements with one irregularity—at element 100—in the array of basilar membrane stiffnesses $K_{1,j}$, while the damping parameters all have the same value (0.4). **a** Frequency spectra for the last 50 ms of the calculated 100 ms of the displacements $x_j(t)$ in a 3D-plot, for oscillator 1 (yellow line at 3.08 kHz), and for elements 2 to 185. The spectra around element 100 are truncated to better show the nodes and antinodes of the standing wave. **b** RMS values for the last 2 ms of the calculated 100 ms of the displacements $x_j(t)$. **c** Detail of the place-to-frequency map for the array of elements, showing the irregularity. **d** Same as panel a (with yellow line at 3.30 kHz). **e** Same as panel b. **f** Same as panel c

**Fig. 19:**
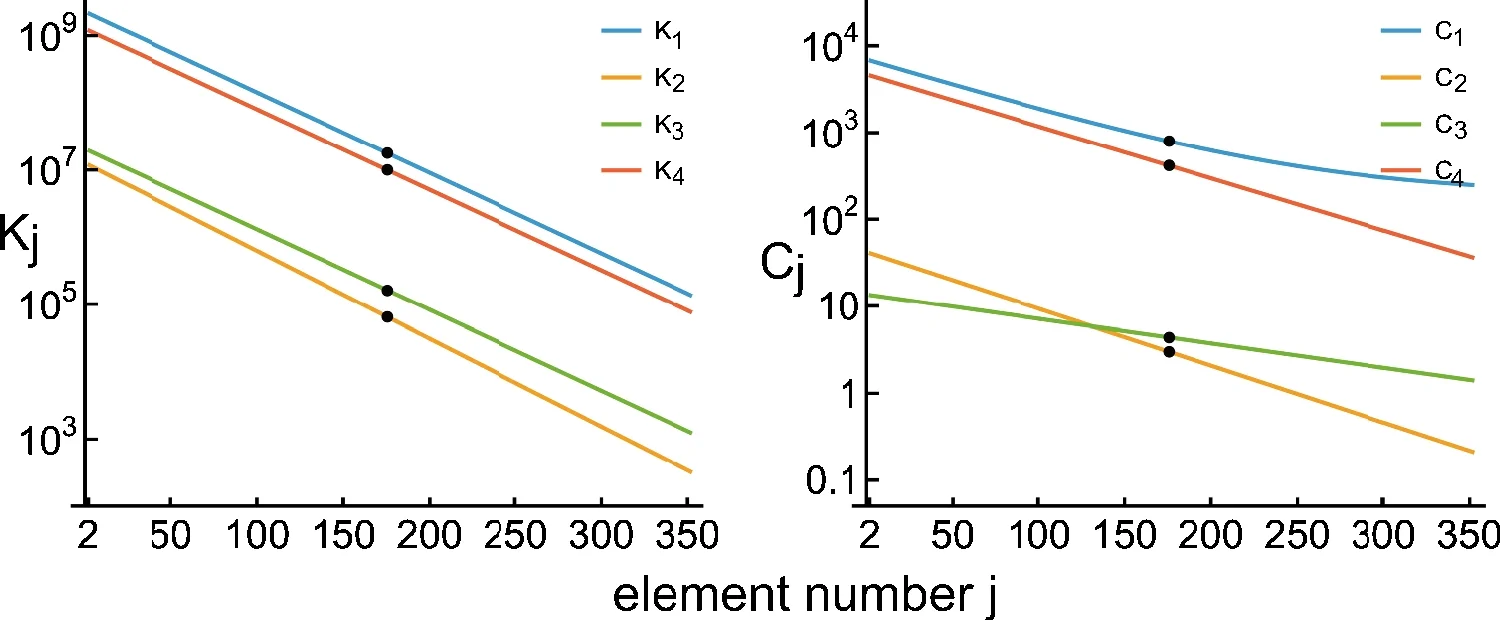
Values for the stiffness parameters $K_j$ and the damping parameters $C_j$ along the array of micromechanical elements in Fig. 2. The black dots mark the values for element 175, as applied in Appendix 1

**Fig. 20:**
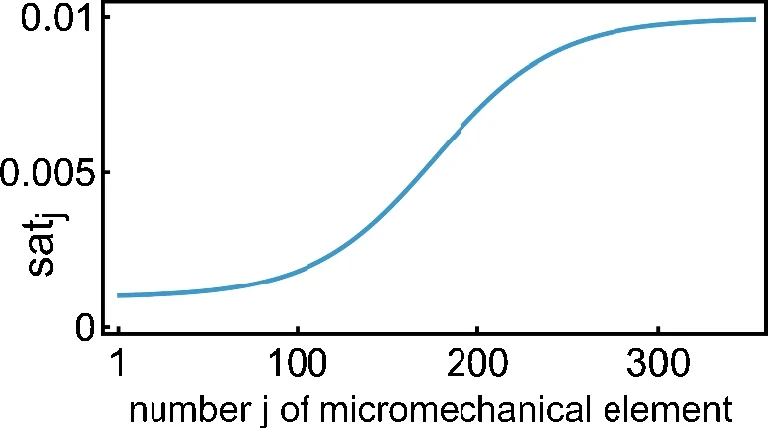
Nonlinear saturation value $sat_j$ (for $s_m=0.001$) for the feedback loops in the micro-mechanical elements

**Fig. 21:**
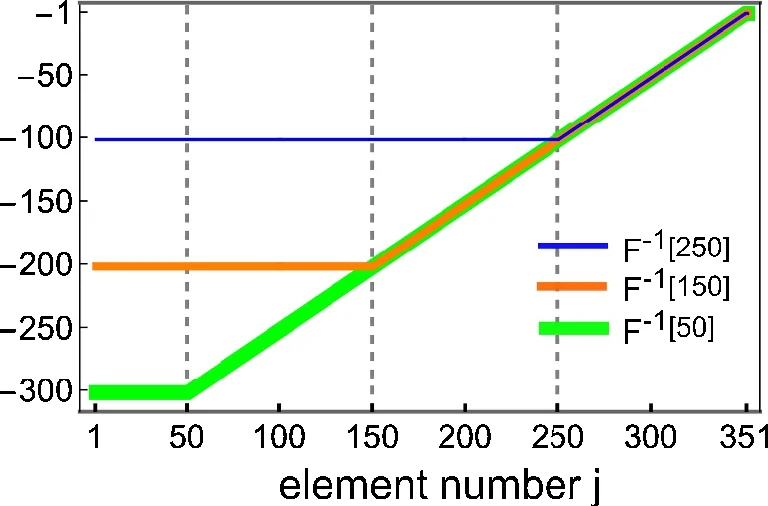
Three rows of inverse matrix $\boldsymbol{F^{-1}}$

## Data Availability

Not applicable.

## Appendix 1. The Neely and Kim Element

The Neely and Kim micro-mechanical cochlear element (Fig. 3 in [35]) consists of two actively coupled harmonic oscillators. Figure 16 shows an adapted version of it, based on figure 3.4 in Vignali’s [18] thesis. Essential in the element is a feedback pressure $P_a$ that compensates for the damping of the two harmonic oscillators (see Fig. 1 in [36] for additional perspective).

Elliott et al. [16] introduced a “saturating nonlinearity” (“nl” in Fig. 16) in Neely and Kim’s cochlear element to prevent that after onset the amplitudes of the two masses $M_1$ and $M_2$ keep increasing if the undamping parameter γ is larger than its threshold value at the bifurcation from positive to negative damping.

The displacements *x*(*t*) and *y*(*t*) of masses $M_1$ and $M_2$ respectively are described by the following set of equations:

4 $$\begin{aligned} {\begin{matrix} M_1\ddot{x}+(C_1+C_3)\dot{x} + (K_1+K_3)x=\gamma \gamma _{nl}(x,y) K_4(x-y)+\gamma \gamma _{nl}(x,y,) C_4(\dot{x} -\dot{y})+K_3y +C_3\dot{y},\\ M_2\ddot{y}+(C_2+C_3)\dot{y} + (K_2+K_3)y=K_3x +C_3\dot{x}, \end{matrix}} \end{aligned}$$

where the nonlinearity is specified as:

5 $$\begin{aligned} \gamma _{nl}(x,y)=1-\tanh [((x-y)/max)^2] \end{aligned}$$

(Adapted from Eqs. 4 and 5 in [37]).

To solve this set of equations, the values for $K_j$ and $C_j$ (j=1,2,3,4) were derived from the formulas in Table 4.1 of Vignali’s thesis [18], for x=17.5 mm. The $K_j, j= 1,2,3,4$ are respectively the stiffness of the basilar membrane, the tectorial membrane, and the passive and active outer hair cell impedances. The $C_j$ are the damping terms in the same order.

The natural frequencies $\sqrt{K_1/M_1}$ and$\sqrt{K_2/M_2}$ are then 3.26 and 0.48 kHz, respectively. Initial values were $x(0)=0, \dot{x}(0)=100, y(0)=0, \dot{y}(0)=100$. Time step: 5μs. And max=1.

Results for two values of damping parameter γ are shown in Fig. 17.

It can be concluded from Fig. 17 that a single Neely and Kim micro-mechanical element, as shown in Fig. 16, can be passive or active, depending on the value of the damping parameter γ, as was already shown by Elliott et al. ([16]; Fig. 12). The element oversteps a Hopf bifurcation if γ is increased from 1.09 to 1.10.

Another example of micro-mechanical elements being two coupled Hopf oscillators is that used by Lerud et al. [38] to model the dynamics of the mammalian cochlea, where they argued that a single oscillator model cannot adequately fit auditory nerve tuning curves. In this system, one oscillator is taken to represent the basilar membrane, while the other represents the Organ of Corti. Results for this system, comparable to Fig. 17 above, are given in [9], Fig. 14.

More information on the behavior of two coupled Hopf-type oscillators can be found in Gomez et al. [39] and in Folke et al. [40].

In Fruth et al.’s active oscillator model [17] to describe the statistics of human spontaneous otoacoustic emissions, the micro-mechanical elements are single Hopf oscillators at one or the other side of their bifurcation, depending on the static spatial variation of the bifurcation parameter, describing cochlear imperfections (see their Fig. 1).

The micro-mechanical elements in a very recently published model, that investigates nonlinear sound processing in the cochlea, are also Hopf oscillators, functioning at their bifurcation [41].

And Alonso et al. have—also very recently [42]—shown that active amplification in a small segment of the mammalian cochlea is achieved by operating near critically at a Hopf bifurcation.

To account for the generation of SOAEs in a very early (1985) model [43], the elements were Van der Pol oscillators, being proposed 5 years earlier by Johannesma [44, 45] to account for the existence of spontaneous emissions. Noticing this, theorem 7.1 in Duifhuis’s book on cochlear mechanics [46] is worth considering: “There is no formal distinction between a properly defined Hopf bifurcation cochlea model and a general Van der Pol oscillator.”

## Appendix 2. Other Irregularities

SOAEs can—for instance—also be modelled by irregularities in the exponential frequency-to-place map for the array of micro-mechanical elements. To illustrate this, the calculations to obtain Fig. 4 were repeated. But now for all damping parameters having the same value 0.4, while a single irregularity was present in the array of basilar membrane stiffnesses $K_{1,j}$: $K_{1,100}$ was given a larger or a smaller value, creating a single irregularity in the frequency-to-place relation. Results for two multiplication factors of $K_{1,100}$ are given in Fig. 18. Figure 18c and f were calculated for all $\gamma _{0,j}$ being 1.35 (all elements being self-sustaining) and no fluid coupling (α=0).

If Fig. 18a, b, d, and e are compared with the corresponding panels in Fig. 4, it is clear that Fig. 18 also shows characteristics of a single global oscillator: a standing wave in a resonant cavity and a single SOAE with the same frequency as the standing wave.

## Appendix 3. Additional Parameter Values and Equations

The values for the stiffness parameters $K_j$ and the damping parameters $C_j$ for each of the elements *j*—as plotted in Fig. 19—were taken from Table 4.1 in [18]. This table also gives formulae and units for the parameters, which can also be found in Table 1 of [16].

The a1i,i=1,...,4 in Eqs. 1 and 2 are:

$$a11_j=g\gamma _{0,j}C_{4,j}\gamma [j.x_j(t)-y_j(t)]-(C_{1,j}+C_{3,j})$$

$$a12_j=g\gamma _{0,j}K_{4,j}\gamma [j,x_j(t)-y_j(t)]-(K_{1,j}+K_{3,j}$$

$$a13_j=C_{3,j}-\gamma _{0,j}C_{4,j})\gamma [j,x_j(t)-y_j(t)]$$

$$a14_j=K_{3,j}-\gamma _{0,j}K_{4,j}\gamma [j,x_j(t)-y_j(t)]$$

with nonlinearity:

$$\gamma (j,x_j(t)-y_j(t))=1-\tanh [((x_j(t)-y_j(t))/sat_j)^2]$$

with $sat_j=4.5s_m(1-e^{0.03(n/2-j})/(1+e^{0.03(n/2-j})+5.5s_m$ (j=1,...,n), as shown in Fig. 20 (to be compared with Fig. 2 in [47]).

And the *a*2*i* are:

$$a21_j=C_{3,j}; a22_j=K_{3,j}; a23_j=-(C_{2,j}+C_{3,j}); a24_j=-(K_{2,j}+K_{3,j})$$

Following equations 10 and 11 in Elliott et al. [16], the relation between the array of n local pressures $$\boldsymbol{p_d}(t)$$ and the array of n accelerations $$\boldsymbol{\ddot{x}}(t)$$,where $$\boldsymbol{p_d}(t)=\{p_{d,1}(t), p_{d,2}(t),...,p_{d,n}(t)\}^T$$ and $$\boldsymbol{\ddot{x}}(t)=\{\ddot{x}_1(t)$$, $$\ddot{x}_2(t),...,$$$$\ddot{x}_n(t)\}^T$$, will be given by

6 $$\begin{aligned} \boldsymbol{F}\boldsymbol{p_d}(t)=\alpha \boldsymbol{\ddot{x}}(t) \end{aligned}$$

in which $\boldsymbol{F}$ is a n×n finite difference matrix and α a multiplication factor proportional to fluid density ρ. Three rows of the inverse matrix $\boldsymbol{F^{-1}}$ are shown in Fig. 21 (to be compared with Fig. 4 in [48]).

To calculate all locally acting $p_{d,j}(t)$, both sides of Eq. 7 are multiplied with $\boldsymbol{F^{-1}}$, giving

7 $$\begin{aligned} \boldsymbol{p_d}(t)=a\boldsymbol{F}^{-1}\boldsymbol{\ddot{x}}(t) \end{aligned}$$

The value for fluid pressure parameter α (= 0.02 $kg/m^2$) in (7) was calculated as in Appendix 1 of [19].

## Declarations

### Use of AI

No AI was used for either manuscript preparation or research purposes.
